# Molecular basis of cell membrane adaptation in daptomycin-resistant *Enterococcus faecalis*

**DOI:** 10.1172/jci.insight.173836

**Published:** 2024-11-22

**Authors:** April H. Nguyen, Truc T. Tran, Diana Panesso, Kara S. Hood, Vinathi Polamraju, Rutan Zhang, Ayesha Khan, William R. Miller, Eugenia Mileykovskaya, Yousif Shamoo, Libin Xu, Heidi Vitrac, Cesar A. Arias

**Affiliations:** 1 Department of Microbiology and Molecular Genetics, University of Texas Health Science Center, Houston, Texas, USA.; 2 Department of Medicine, Division of Infectious Diseases, Houston Methodist Hospital, Texas, USA.; 3 Center for Infectious Diseases, Houston Methodist Research Institute, Houston, Texas, USA.; 4 Department of Medicine, Weill Cornell Medical College, New York, New York, USA.; 5 Molecular Genetics and Antimicrobial Resistance Unit, Universidad El Bosque, Bogota, Colombia.; 6 Department of Medicinal Chemistry, University of Washington, Seattle, Washington, USA.; 7 Department of Pathology, Microbiology, Immunology, Vanderbilt University, Nashville, Tennessee, USA.; 8 Department of Biochemistry and Molecular Biology, University of Texas Health Science Center, Houston, Texas, USA.; 9 Department of Biosciences, Rice University, Houston, Texas, USA.; 10 Bruker Daltonics, Los Angeles, California, USA.

**Keywords:** Infectious disease, Bacterial infections, Lipid rafts, Peptides

## Abstract

Daptomycin is a last-resort lipopeptide antibiotic that disrupts cell membrane (CM) and peptidoglycan homeostasis. *Enterococcus faecalis* has developed a sophisticated mechanism to avoid daptomycin killing by redistributing CM anionic phospholipids away from the septum. The CM changes are orchestrated by a 3-component regulatory system, designated LiaFSR, with a possible contribution of cardiolipin synthase (Cls). However, the mechanism by which LiaFSR controls the CM response and the role of Cls are unknown. Here, we show that cardiolipin synthase activity is essential for anionic phospholipid redistribution and daptomycin resistance since deletion of the 2 genes (*cls1* and *cls2*) encoding Cls abolished CM remodeling. We identified LiaY, a transmembrane protein regulated by LiaFSR, and Cls1 as important mediators of CM remodeling required for redistribution of anionic phospholipid microdomains. Together, our insights provide a mechanistic framework on the enterococcal response to cell envelope antibiotics that could be exploited therapeutically.

## Abstract 2

Daptomycin is a cell-envelope targeting antibiotic used against multi-drug resistant enterococci. Here, we identify the mechanistic basis of cell membrane remodeling associated with daptomycin resistance.

## Graphical abstract

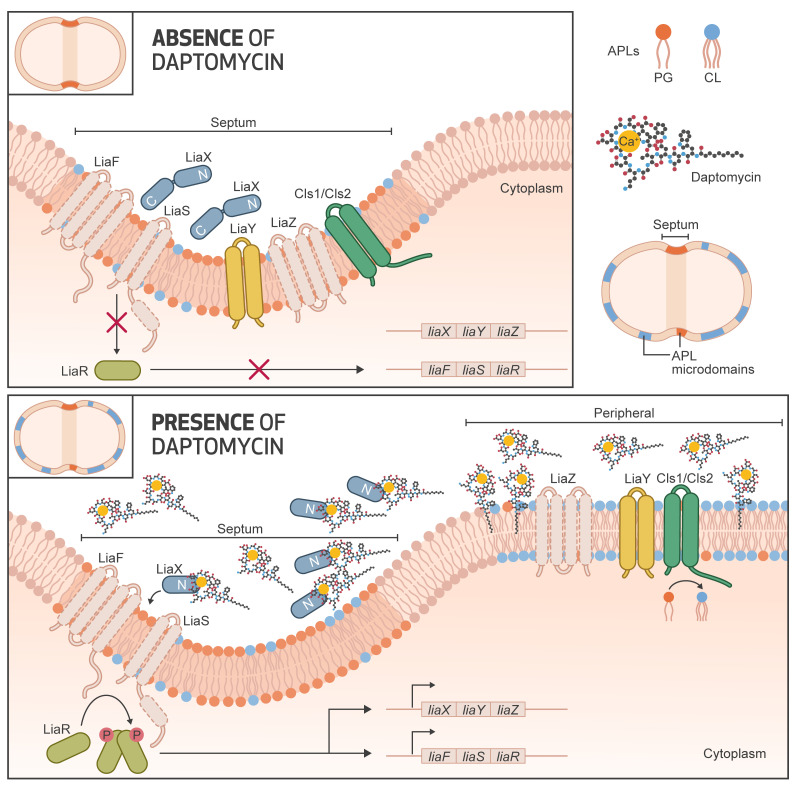

## Introduction

Enterococci are commensal bacteria that cause recalcitrant hospital-associated infections, and effective treatment has become a major challenge due to rising rates of antimicrobial resistance. Daptomycin (DAP) is a lipopeptide antibiotic with in vitro bactericidal activity against enterococci. While the mechanism of action of DAP is not fully understood, DAP is thought to bind to the cell membrane (CM) in a complex with anionic phospholipids (APLs) such as phosphatidylglycerol (PG) at the division septum (1–3). DAP is then thought to insert into the membrane in complex with calcium, lipid II, and PG. Interactions with CM lipid also appear to displace key proteins such MurG and PlsX, disrupting CM homeostasis and cell wall metabolism, ultimately leading to bacterial death (1, 2).

In *Enterococcus faecalis* (*Efs*), DAP-resistance (DAP-R) is mediated by LiaFSR, a 3-component cell envelope stress response system. Activation of the system causes CM changes, including alterations in lipid content and localization of APL microdomains, a hallmark of DAP-R (3–7). Previously, we characterized LiaX, an effector of the LiaFSR CM response, and showed that its C-terminal regulates the CM response against DAP through an unknown mechanism (4). DAP-R has also been associated with mutations in cardiolipin synthase (Cls), which synthesizes cardiolipin (CL) using PG as substrate (5, 8, 9). CL content appears to affect several CM functions, including cell division (10–13), and previous studies have linked increased levels of CL to DAP-R in *Efs* and *Staphylococcus aureus* (13–17). *Efs* harbors 2 *cls* genes (*cls1* and *cls2*), with the majority of mutations associated with DAP-R identified in *cls1* (5, 8, 9). Indeed, an R218Q substitution in the putative catalytic domain of Cls1 resulted in gain-of-function activity (15). Experimental evolution of a DAP-susceptible (DAP-S) strain of *Efs* under DAP exposure has shown that Cls substitutions commonly arise following changes in LiaFSR, leading to high-level DAP-R (9), suggesting that Cls may function in conjunction with LiaFSR to modulate CM adaptation. Here, we aimed to characterize the mechanistic basis of LiaFSR and Cls-mediated changes in CM adaptation that lead to DAP-R. We identified a potentially novel transmembrane protein (designated LiaY) that, in conjunction with Cls, bridges the gap between recognition of the antibiotic (DAP) cell envelope attack and major changes in phospholipid architecture resulting in the development of antimicrobial peptide resistance.

## Results

### The cls genes have overlapping functions and are required for APL redistribution in DAP resistance.

Both Cls1 and Cls2 synthesize CL (13); however, DAP-R mutations have been identified primarily in *cls1* (5, 8, 9). Since Cls1 may function with the LiaFSR system to modulate the CM adaptive response (9), we initially evaluated how activation of LiaFSR affects the expression of *cls1* and *cls2* using a DAP-R strain that lacks *liaX* (*Efs* OG117Δ*liaX*) (4, 18). Of note, deletion of *liaX* or the region coding for the C-terminus constitutively activates the LiaFSR system and results in DAP-R (4).

**Figure 1:**
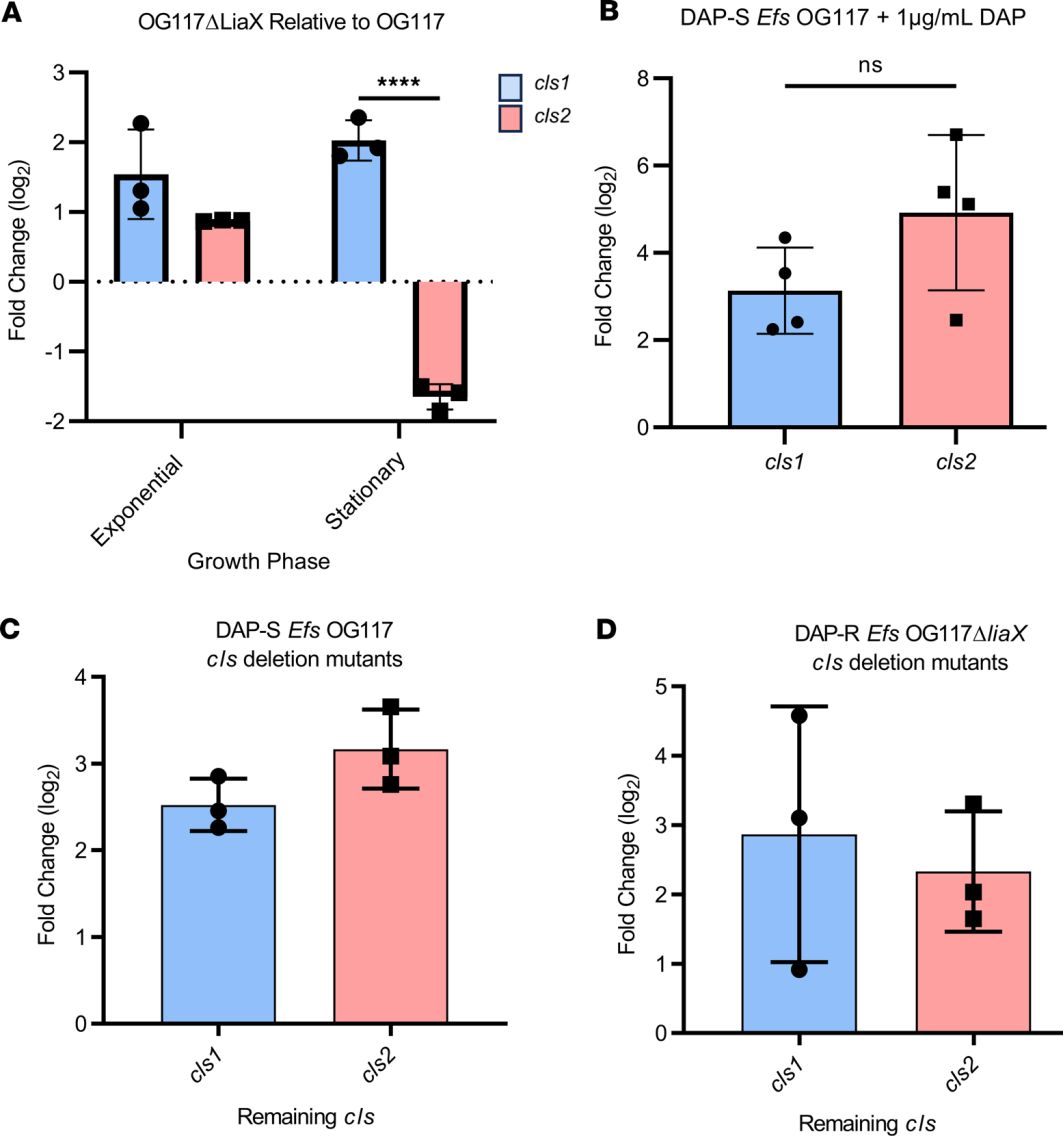
The *cls* genes are upregulated in association with DAP and can compensate for each other. (**A**) qPCR results evaluating *cls1* and *cls2* expression in DAP-R *Efs* OG117Δ*liaX* relative to DAP-S *Efs* OG117. ****P < 0.0001, *n* = 3–4 replicates, individual *t* test. (**B**) qPCR results evaluating *cls1* and *cls2* expression in DAP-S *Efs* OG117 with DAP 1 μg/mL relative to DAP-S *Efs* OG117. *n* > 3 replicates via unpaired 2-tailed *t* test. (**C**) *cls1* or *cls2* gene expression in *Efs* OG117Δcls1 and *Efs* OG117Δcls2 relative to *Efs* OG117. *n* = 3, unpaired 2-tailed *t* test. (**D**) *cls1* or *cls2* gene expression in *Efs* OG117Δ*liaX*Δ*cls1* or *Efs* OG117Δ*liaX*Δ*cls2* relative to *Efs* OG117Δ*liaX*; *n* = 3, unpaired 2-tailed *t* test.

Quantitative PCR (qPCR) (19) showed that *cls1* expression was increased in DAP-R *Efs* OG117Δ*liaX* relative to its DAP-S parental strain (*Efs* OG117). In contrast, while *cls2* expression was increased in exponential phase growth, there was a sharp decrease in *cls2* expression in the stationary phase in the DAP-R strain (Figure 1A). These results suggest that the dynamics of *cls* gene expression differ between *cls1* and *cls2* within the cell cycle, with *Cls1* playing a more prominent role during stationary phase. We also tested the activation of *cls* genes in a DAP-S strain upon exposure to a subinhibitory concentration of DAP. Figure 1B shows that both *cls1* and *cls2* are upregulated upon membrane sensing of DAP. To test whether *cls1* and *cls2* were able to compensate for each other, we evaluated their expression in mutants containing individual deletions of either *cls1* or *cls2* in both *Efs* OG117 and *Efs* OG117Δ*liaX*. Figure 1, C and D, shows that, independent of the genetic background, deletion of either *cls* ultimately results in upregulation of the remaining *cls*, supporting functional redundancy between them.

**Figure 2:**
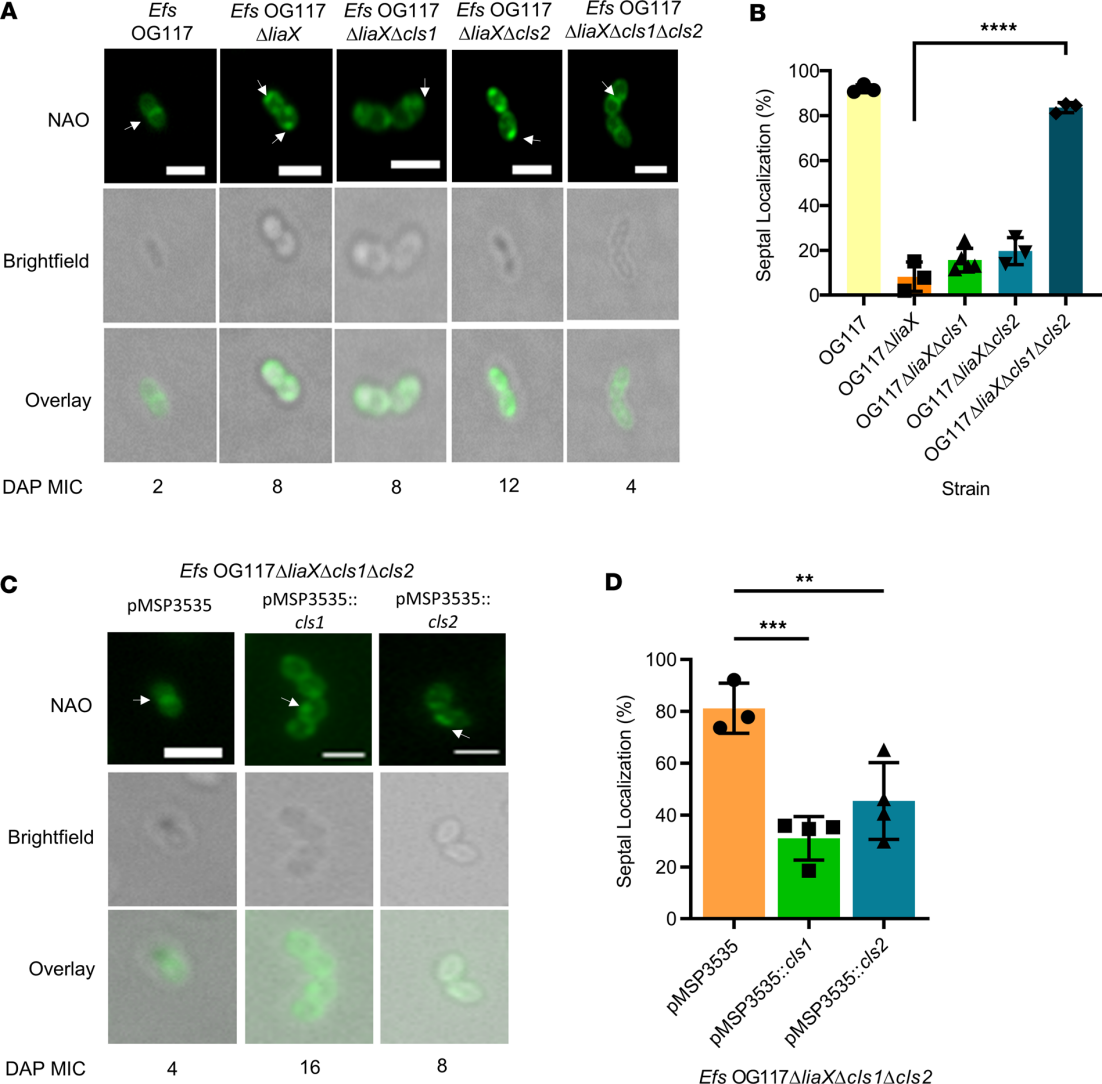
Both *cls* genes are required for DAP-R anionic phospholipid redistribution phenotype. (**A**) Representative images of NAO staining (top row), bright-field (middle row), and overlay (bottom row) in cls mutant derivatives of *Efs* OG117Δ*liaX* and DAP MICs (under images). Scale bar: 2μm. White arrows represent anionic phospholipid microdomains at mid-cell or non–mid-cell locations. (**B**) Quantification of septal localization of anionic phospholipid microdomains with NAO in cls mutant derivatives of *Efs* OG117Δ*liaX* by counting a minimum of 50 cells per replicate (*n* = 3–5 replicates, ****P < 0.0001, 1-way ANOVA with multiple comparisons). (**C**) Representative images of NAO staining (top row), bright-field (middle row), and overlay (bottom row) in *cls* mutant derivatives of *Efs* OG117Δ*liaX* complemented with vector pMSP3535 and derivatives with DAP MICs (under images). White arrows represent anionic phospholipid microdomains at mid-cell or non–mid-cell locations. Scale bar: 2 µm. (**D**) Quantification of septal localization of anionic phospholipid microdomains with NAO in cls mutants of *Efs* OG117Δ*liaX* complemented with vector pMSP3535 and derivatives by counting a minimum of 50 cells per replicate. *n* = 3–4, **P < 0.01, ***P < 0.001, 1 way ANOVA with multiple comparisons. Whole images were adjusted for “Black Balance” per BZ-X800 Image Analysis Software with individual representative selected.

Deletion of *cls1* or *cls2* individually did not alter DAP-associated phenotypes in the DAP-S *Efs* OG117. Indeed, the DAP minimum inhibitory concentration (MIC) remained within susceptible levels (1–1.5 μg/mL). The mutants were also characterized through localization of their APL microdomains stained with 10-N-nonyl acridine orange (NAO). Our previous work (4, 6) has shown that APL microdomains localize at the mid-cell, representing the septum (or sites of negative curvature — i.e., forming of previous septa at the poles) in DAP-S strains of *Efs* and away from the mid-cell in DAP-R strains. Using colocalization of NAO with HCC-Amino-D-alanine hydrochloride (HADA, a fluorescent dye that binds peptidoglycan, representative of the division septum), we show that these microdomains were indeed redistributed away from the division septum upon development of DAP resistance (Supplemental Figure 1; supplemental material available online with this article; https://doi.org/10.1172/jci.insight.173836DS1). Furthermore, APL microdomains in DAP-S *Efs* OG117 with deletions of *cls1* and/or *cls2* exhibited WT septal localization (Supplemental Figure 2A). Similarly, individual deletions of either *cls1* or *cls2* in DAP-R OG117Δ*liaX* did not alter the DAP MIC or APL microdomain localization (nonseptal pattern), with strains remaining DAP-R (8–12 μg/mL; Figure 2A, Supplemental Figure 3, and Supplemental Table 1). These findings were confirmed through quantification of a subset of cells (*n* = 50) to determine whether fluorescence intensity was concentrated at the mid-cell or non–mid-cell areas as indicated by arrows (Figure 2B and Supplemental Figure 2B).

We next generated tandem deletions of *cls1* and *cls2* to determine the effect of the complete absence of *cls* genes. Similar to single *cls* deletions, double deletions of both *cls* genes in DAP-S OG117 did not alter DAP MIC or APL localization (Supplemental Figure 2). In contrast, a double deletion of both *cls1* and *cls2* in DAP-R *Efs* OG117Δ*liaX* reverted the resistance phenotype and restored septal localization of APL microdomains (Figure 2, A and B; Supplemental Figure 3; and Supplemental Table 1).

To confirm the specific role of the *cls* genes in membrane adaptation, we *trans*-complemented the *Efs* OG117Δ*liaX*Δ*cls1*Δ*cls2* mutant with *cls1* and *cls2* genes individually, using a nisin-induced plasmid (pMSP3535) (20). Expression of either *cls1* or *cls2* from pMSP3535 increased the DAP MICs and restored the nonseptal APL microdomain localization (Figure 2, C and D; Supplemental Table 1; and Supplemental Figure 4). We also cloned *cls1* and *cls2* independently in pAT392 under the constitutive promoter P2 (21). Constitutive expression of *cls1* in *Efs* OG117Δ*liaX*Δ*cls1*Δ*cls2* also increased the DAP MIC and reverted the septal localization of APL microdomains (Supplemental Figure 5). Of note, constitutive expression of *cls2* using pAT392 in *Efs* OG117Δ*liaX*Δ*cls1*Δ*cls2* resulted in a clumping phenotype with an unclear pattern of APL microdomain localization (Supplemental Figure 5A). However, DAP MIC was still increased (Supplemental Table 1). Overall, our results suggest a major role for Cls1 in CM adaptation and indicate that CL is the main phospholipid species required for changes in membrane architecture that result in DAP-R.

### CM phospholipid changes associated with DAP-R and deletion of cls genes.

To better understand the biochemical effects of *cls* deletions, we performed membrane lipid analysis using hydrophilic interaction liquid chromatography–ion mobility–mass spectrometry (HILIC-IM-MS) (22, 23) on stationary phase cells, where changes in *cls* expression and CL content have been documented in the context of DAP-R (4). As CL is a terminal product of a phospholipid biosynthetic pathway, changes in *cls* may trigger alterations in other lipid classes within the same or interrelated pathways (i.e,. PG, lysyl-PG, diacylglycerol [DG], diglycosyl diacylglycerol [DGDG]; refs. 4, 13, 22, 24, 25).

**Figure 3:**
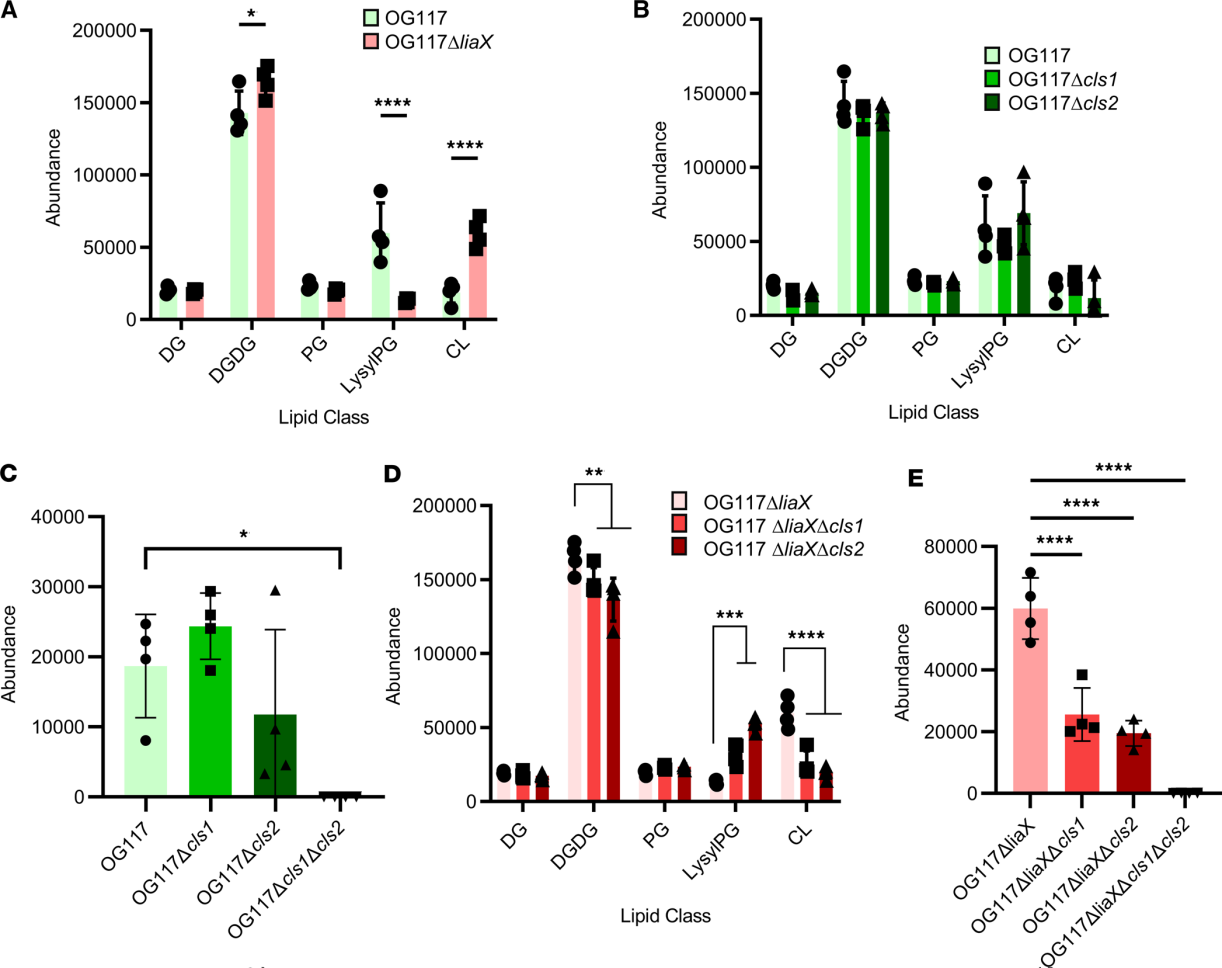
Cell membrane phospholipid changes associated with DAP-R and deletion of cls genes. (**A**) Quantification of lipid classes in DAP-susceptible *E. faecalis* OG117 and DAP-R *E. faecalis* OG117Δ*liaX*. (**B**) Quantification of lipid classes in DAP-susceptible *E. faecalis* OG117 (parental) and *cls* mutant derivatives. (**C**) Quantification of cardiolipin (CL) content in DAP-susceptible *E. faecalis* OG117 and *cls* mutant derivatives. (**D**) Quantification of lipid classes in DAP-R *E. faecalis* OG117Δ*liaX* and *cls* mutant derivatives. (**E**) Quantification of cardiolipin (CL) content in DAP-R *E. faecalis* OG117Δ*liaX* and *cls* mutant derivatives. DG, diacylglycerol; DGDG, diglycosyldiacylglycerol; PG, phosphatidylglycerol; LysylPG, lysyl-phosphatidylglycerol; CL, cardiolipin. *n* = 4, *P < 0.05, **P < 0.01, ****P < 0.0001, 1- or 2-way ANOVA with multiple comparisons

We first standardized lipid content of our representative DAP-S and DAP-R strains (*Efs* OG117 versus *Efs* OG117Δ*liaX*) under standard growth conditions, depicting the lipid content as abundance of each lipid class per strain. The results indicate that, compared with DAP-S *Efs* OG117, DAP-R *Efs* OG117Δ*liaX* exhibited an increase in CL content, concomitant with a decrease in lysyl-PG and no significant difference in PG content (Figure 3A). We also identified the type and levels of individual CL species present within each strain by the length of fatty acid chain and degree of unsaturation. DAP-R OG117Δ*liaX* exhibited increased levels of CL species containing longer-chain fatty acids and higher unsaturation compared with DAP-S OG117 strain (Table 1).

Individual deletions of either *cls1* or *cls2 in* DAP-S OG117 did not alter the overall content of CL or any other lipid classes tested compared with the parental strain. Importantly, there were also no differences in overall CL levels when comparing the *cls1* versus *cls2* deletion in DAP-S OG117 (Figure 3B). Individual deletions of *cls1* or *cls2* caused a shift in CL species, favoring fatty acids with longer chain length and higher degree of unsaturation compared with the parental *Efs* OG117 (Table 2). Of note, individual *cls* deletions showed no differences in CL species content compared with each other, further supporting the redundancy of their functions (Table 2). Most importantly, our lipidomics analyses confirmed that deleting both *cls1* and *cls2* completely abolished CL content in the membrane of DAP-S *Efs* OG117, demonstrating that Cls1 and Cls2 are true CL synthases (13) (Figure 3C). Interestingly, deletion of CL from the membrane in the DAP-S background (double *cls* mutant; OG117Δ*cls1cls2*) led to global changes in lipid content, including increases in PG and lysyl-PG (Supplemental Figure 6).

CM content analyses of DAP-R *Efs* OG117Δ*liaX* showed that individual *cls* deletions yielded a decrease in DGDG and CL content, as well as an increase in lysyl-PG content (Figure 3D). Furthermore, the overall amount of CL produced by DAP-R *Efs* OG117Δ*liaX*Δ*cls1* or *Efs* OG117Δ*liaX*Δ*cls2* was very similar to that of DAP-S *Efs* OG117Δ*cls1* and *Efs* OG117Δ*cls2* (Figure 3, C and E). However, the CL species produced by DAP-R *Efs* OG117Δ*liaX*Δ*cls1* and *Efs* OG117Δ*liaX*Δ*cls2* harbored shorter chain fatty acids and lower degree of unsaturation when compared with the parent *Efs* OG117Δ*liaX* (Table 3). Nonetheless, when comparing individual *cls* deletions between each other in this DAP-R background, we found no statistically significant differences in levels of CL species except for CL 64:2, 65:2, 70:3, and 71:3 (Table 3). Finally, similar to OG117, deletion of both *cls1* and *cls2* in DAP-R *Efs* OG117Δ*liaX* resulted in total abolishment of CM CL content (Figure 3E). In a recapitulation of the DAP-S background, double *cls* deletion caused increased lysyl-PG but with additional changes including decreased DGDG content with no major changes to PG levels (Supplemental Figure 6)

Put together, our results suggest that CL plays a major role in DAP-R and that the phenotype results in increased CL content with longer fatty acids and a higher degree of unsaturation. This biochemical signature appears to be mediated by contributions and overlapping functions of both Cls1 and Cls2.

### Cls1 localizes in nonseptal APL microdomains in DAP-R Efs.

**Figure 4:**
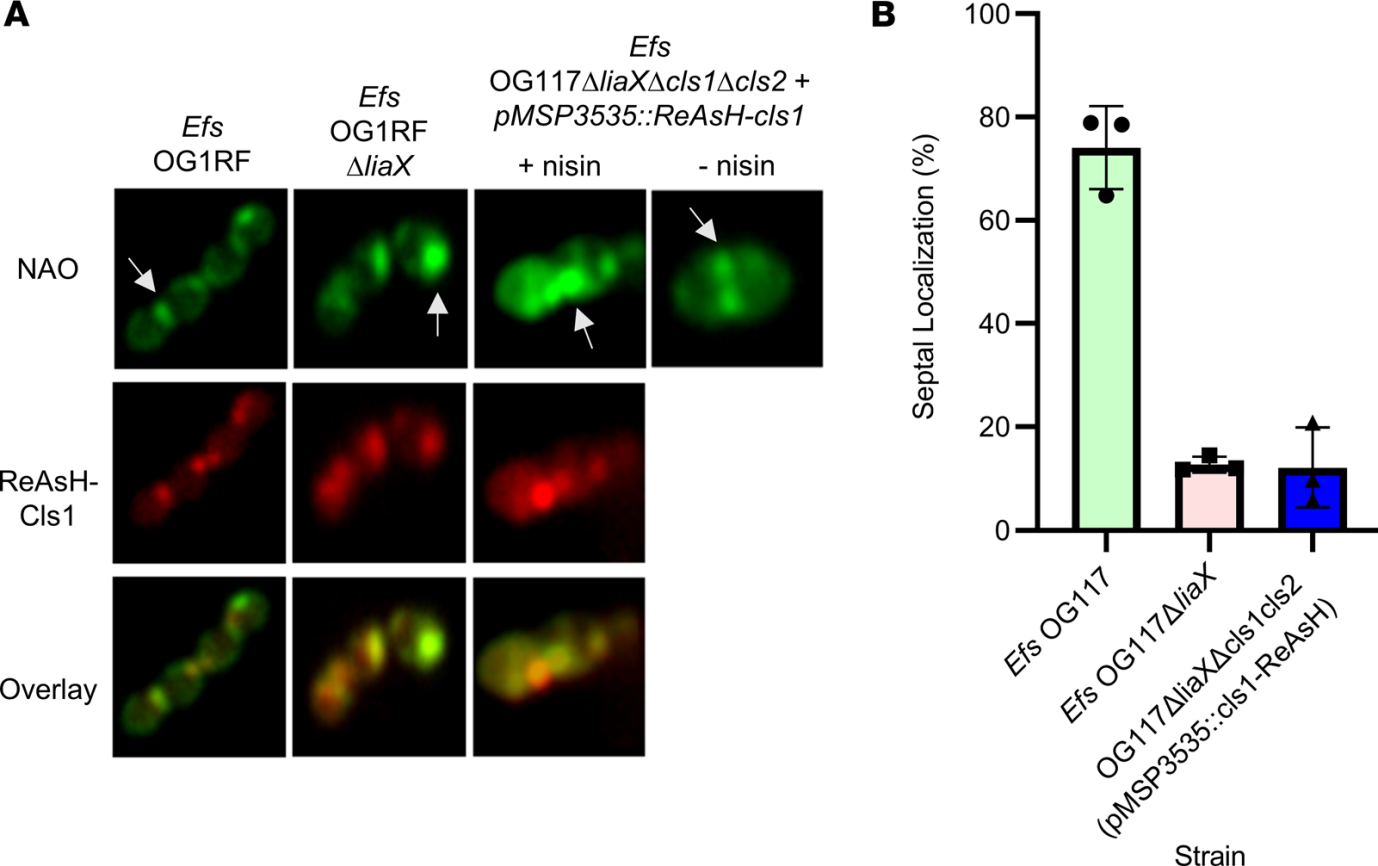
Cls1 localizes in nonseptal anionic phospholipid microdomains in DAP-R *E. faecalis*. (**A**) Left 2 panels: Representative images of NAO staining (top panel), tetracysteine-tagged Cls1 with ReAsH reagent (red fluorescence, middle panel), and overlay of both images (bottom panel). White arrows represent anionic phospholipid microdomains at mid-cell or non–mid-cell locations. Both anionic phospholipid microdomains and Cls1 are shown to relocalize and overlap away from the septum in DAP-R *Efs* (OG1RFΔ*liaX*) (Pearson Correlation Coefficient, 0.91 [95% CI, 0.74–0.97]) compared with their septal pattern in DAP-S *E. faecalis* OG1RF (Pearson Correlation Coefficient, 0.94 [95% CI, 0.83–0.98]). Right 2 panels: NAO staining for anionic phospholipid microdomains (green) and tetracysteine tagged-Cls1 localization (red) in *E. faecalis* OG117Δ*liaX*Δ*cls1*Δ*cls2* harboring pMSP3535 carrying the gene expressing the tetracysteine tagged-Cls1 with or without nisin induction at 50 ng/mL, overlap between NAO and ReAsH (Pearson Correlation Coefficient, 0.91 [95% CI, 0.76–0.96]). White arrows represent anionic phospholipid microdomains at mid-cell or non–mid-cell locations. Whole images were adjusted for “Black Balance” per BZ-X800 Image Analysis Software with individual representative selected. (**B**) Quantification of Cls septal localization for strains, minimum 50 cells counted/strain, *n* = 3.

Our initial results provided evidence that the formation of nonseptal APL microdomains is dependent on the availability of CL and CL synthases. Thus, we hypothesized that Cls1 is likely responsible for the formation of nonseptally located APL microdomains. Therefore, we evaluated the membrane localization of a tetracysteine-tagged Cls1 by fluorescence microscopy using a ReAsH biarsenical reagent (26) coupled with NAO staining in both DAP-S *Efs* OG1RF and DAP-R *Efs* OG1RFΔ*liaX*. Figure 4 shows that, (a) in DAP-S *Efs* OG1RF, Cls1 is localized at the septum; (b) in DAP-R *Efs* OG1RFΔ*liaX*, Cls1 is localized away from the septum; and (c) Cls1 was colocalized with APL microdomains in both conditions (Figure 4 and Supplemental Figure 7). To confirm the colocalization of Cls1 and APL microdomains, we cloned the gene expressing ReAsH-Cls1 in the nisin-induced pMSP3535 and transformed it into OG117Δ*liaX*Δ*cls1*Δ*cls2*. Figure 4 shows that, in the absence of nisin induction, APL microdomains are mainly visualized at the septum in the *cls* double-deletion strain OG117Δ*liaX*Δ*cls1*Δ*cls2*. Nisin induction of pMSP35335:*ReAsH-cls1* restored the nonseptal localization of the APL microdomains, confirming its essentiality in mediating CM remodeling in DAP-R (Figure 4 and Supplemental Figure 8).

Furthermore, since we previously showed here that Cls1 and Cls2 have redundant functions in DAP-R, we also investigated the localization dynamics of a GFP-tagged Cls2 in both DAP-S and DAP-R strains. Indeed, DAP-S *Efs* OG117Δ*cls1* and DAP-R OG117Δ*liaX*Δ*cls1* exhibited septal and nonseptal localization of Cls2, respectively (Supplemental Figure 9). Taken together, our results highlight the major role of Cls in triggering changes in membrane architecture mediating DAP-R.

### LiaY, a member of the LiaFSR system, mediates CM remodeling associated with DAP-R.

**Figure 5:**
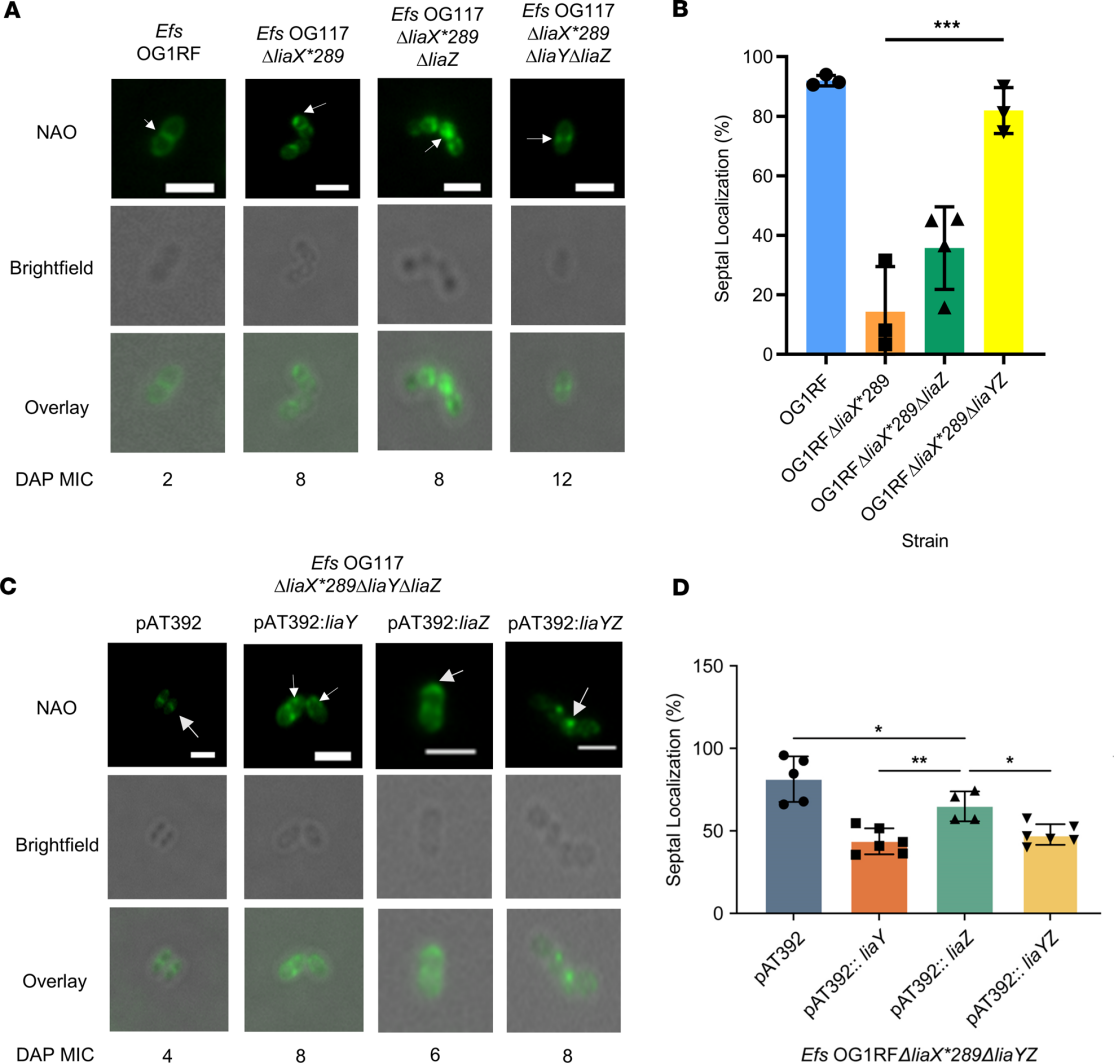
LiaY, a member of the LiaFSR system, mediates changes in phospholipid architecture associated with DAP resistance. (**A**) Representative images of anionic phospholipid localization by NAO staining (top row), bright-field (middle row), and overlay (bottom row) in DAP-R *Efs* OG1RFΔliaX*289 and *liaYZ* mutants. DAP MICs (under images). White arrows represent anionic phospholipid microdomains at mid-cell or non–mid-cell locations. Scale bar: 2 μM. (**B**) Quantification of septal localization of anionic phospholipid microdomains by NAO staining in *Efs* OG1RF and *liaXYZ* mutants from **A** (>50 cells counted per strain per replicate, *n* = 3–4 replicates). ***P < 0.001 via 1-way ANOVA with multiple comparisons. (**C**) Representative images of anionic phospholipid localization by NAO staining (top row), bright-field (middle row), and overlay (bottom row) in *Efs* OG1RF liaX*289ΔliaYZ mutant transformed with pAT392 and derivatives. DAP MICs (under images). White arrows represent anionic phospholipid microdomains at mid-cell or non–mid-cell locations. Scale bar: 2 μM. (**D**) Quantification of septal localization of anionic phospholipid microdomains by NAO staining of *Efs* OG1RF liaX*289Δ*liaYZ* mutant complemented with pAT392 and derivatives from **C** (>50 cells counted per strain per replicate, *n* = 6–9 replicates). *P < 0.05; **P < 0.001, 1-way ANOVA with multiple comparisons. Whole images were adjusted for “Black Balance” per BZ-X800 Image Analysis Software with individual representative selected.

The main target of the LiaR response regulator is a 3-gene cluster designated *liaXYZ*. We previously showed that LiaX plays a major role in controlling the activation of the CM response through its C-terminal domain (4). Here, we sought to characterize the role of *liaYZ* in DAP-associated CM remodeling. In silico evaluation indicated that LiaY is a 107–amino acid transmembrane protein that is predicted to contain a PspC domain (involved in the phage shock protein stress response in *E*. *coli*) (27). LiaZ is a 118–amino acid transmembrane protein that shows homology with holins, generally involved in autolysis (28). We initially attempted to generate KO mutants (29, 30) of *liaY*, *liaZ*, and both genes in the background of DAP-R *Efs* OG1RF liaX*289 (harboring a deletion of the region encoding the C-terminus of LiaX; ref. 4). In *Efs* OG1RF liaX*289, deletion of *liaZ* had a marginal effect on DAP MICs (decreasing 1.5-fold) and the mutant predominantly exhibited a nonseptal pattern of APL microdomains (Figure 5, A and B). We were unable to delete *liaY* in *Efs* OG1RF liaX*289 without generating compensatory mutations in *liaS* (Supplemental Table 3). In contrast, we successfully generated individual *liaY* mutants in DAP-S OG1RF without additional mutations in *liaFSR*. Of note, characterization of these *liaY* mutant strains indicated that deletion of *liaY* in DAP-S OG1RF (without an activated LiaFSR system) did not affect the DAP MIC. While there was a decrease in septal localization relative to WT when *liaYZ* was deleted, the overall APL microdomain localization remained predominantly at the mid-cell (Supplemental Figure 10), supporting the notion that LiaY is critical mainly when the LiaFSR is activated to maintain the DAP-R phenotype.

Despite difficulties in generating *liaY* mutants in DAP-R *Efs* OG1RF liaX*289, we were able to make a double mutant of *liaYZ*. The double mutant had a reduced DAP MIC (6 μg/mL compared with 12 μg/mL in the parent strain) (Supplemental Table 1) with restoration of septal APL microdomain localization (Figure 5, A and B, and Supplemental Figure 11). These findings were consistent with our hypothesis that *liaY* was important in the generation of nonseptal phospholipid microdomains associated with DAP-R.

To confirm the role of *liaY* in CM remodeling without specifically generating a *liaY* deletion, we opted to introduce *liaY*, *liaZ*, or *liaYZ* into the double-mutant *Efs* OG1RF liaX*289Δ*liaYZ* using pMSP3535 (nisin inducible) (20) and pAT392 (21). Transcomplementation of *Efs* OG1RF liaX*289Δ*liaYZ* with *liaY* on pMSP3535 (Supplemental Figure 12) or pAT392 (Figure 5, C and D, and Supplemental Figure 13) caused an increase in DAP MICs in OG1RF liaX*289Δ*liaYZ* (3- and 2-fold, respectively) (Supplemental Table 1). We also identified an increase in the nonseptal localization of APL microdomains, confirming the role of LiaY in DAP-mediated CM adaptation. Constitutive expression of *liaZ* in *Efs* OG1RF liaX*289Δ*liaYZ* also caused an increase in DAP MIC. However, the effect on APL microdomain localization was less pronounced compared with *trans* expression of *liaY* or *liaYZ* (Figure 5, C and D, and Supplemental Figure 13). Of note, introduction of *liaZ* under the constitutive promoter P2 in pAT392 resulted in a proportion of the population with a clumping phenotype similar to that observed with *cls2* expression in OG117Δ*liaX*Δ*cls1*Δ*cls2* (Supplemental Figures 13 and 14). Thus, our results indicate that both LiaY and LiaZ contribute to the redistribution of APL microdomains associated with DAP-R, although LiaY seems to play the more dominant role in this effect.

### LiaY bridges the LiaFSR response with changes in membrane architecture via Cls.

**Figure 6:**
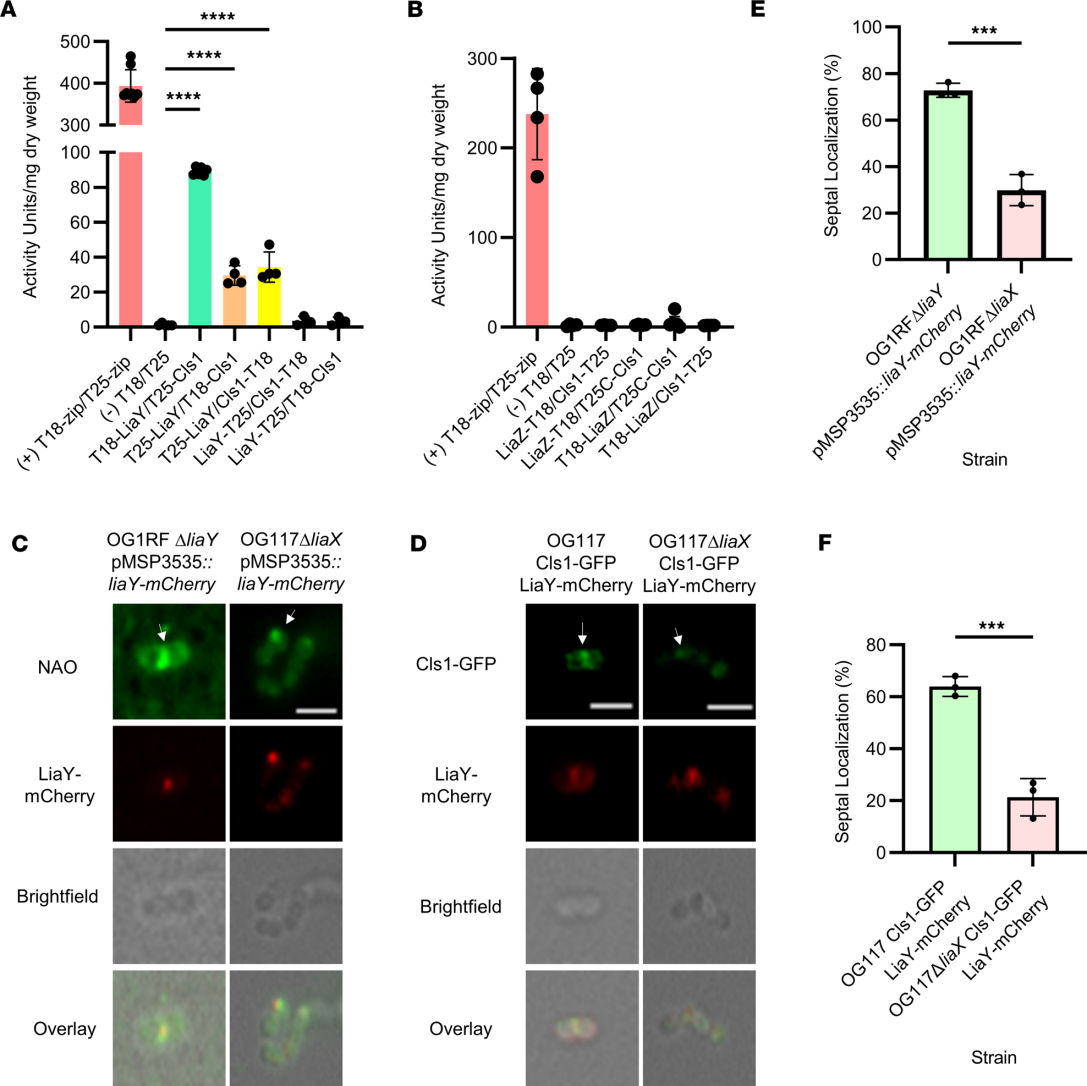
LiaY bridges the LiaFSR response with changes in membrane architecture via Cls. (**A**) Protein-protein interactions between LiaY and Cls1 using the bacterial 2-hybrid system. Proteins were tagged at either the N- or C-terminus, cotransformed into *E. coli* BTH101, and activity recorded via a β-galactosidase assay. A leucine zipper interaction was used as the positive control (T18-zip/T25-zip, red bar) with 2 nontagged empty vectors used as negative controls (T18/T25). *n* = 3–8, ****P < 0.001, 1-way ANOVA with multiple comparisons with (-) against LiaY/Cls1 combinations. (**B**) Protein-to-protein interactions between LiaZ and Cls1 using the bacterial 2-hybrid system and following the same methodology as in **A**. *n* = 4–7. (**C**) Representative images of NAO staining (top row), mCherry (second row), bright-field (third row), and overlay (bottom row) in DAP-S (*Efs* OG1RFΔ*liaY*) (Pearson Correlation Coefficient, 0.67 [95% CI, 0.24–0.88]) and DAP-R (*Efs* OG117ΔliaX) (Pearson Correlation Coefficient, 0.91 [95% CI, 0.75–0.97]). Both strains were transformed with pMSP3535::*liaY-mCherry*. White arrows represent anionic phospholipid microdomains at mid-cell or non–mid-cell locations. Scale bar: 2 μM. (**D**) Representative images of Cls1-GFP (top row), LiaY-mCherry (second row), bright-field (third row), and overlay (bottom row) of LiaY-mCherry and Cls1-GFP via fluorescence microscopy in *Efs* OG117 (Pearson Correlation Coefficient, 0.69 [95% CI, 0.28–0.89]) and *Efs* OG117Δ*liaX* GFP-Cls1 (Pearson Correlation Coefficient, 0.86 [95% CI, 0.63–0.95]) (with *gfp-cls1* introduced in the chromosome) transformed with pMSP3535::*liaY-mCherry*. White arrows represent anionic phospholipid microdomains at mid-cell or non–mid-cell locations. Scale bar: 2 μM. Whole images were adjusted for “Black Balance” per BZ-X800 Image Analysis Software with individual representative selected. (**E** and **F**) Quantification of septal localization, minimum 50 cells counted per strain. *n* = 3, ***P < 0.001, 2-tailed *t* test.

Since both Cls and LiaY are crucial for the formation of nonseptal APL microdomains associated with DAP-R, we investigated if this adaptation was mediated by interactions of LiaY with Cls1 and evaluated this possibility by applying the bacterial 2-hybrid system as an initial screen. LiaY and Cls were tagged either at the N- or C-terminus of adenylate cyclase reporter domains with β-galactosidase activity as a measure of potential interaction. Figure 6A shows that the LiaY and Cls1 exhibited a likely interaction. Of note, we were not able to detect a potential interaction between Cls1 and LiaZ (Figure 6B). We next sought to evaluate colocalization of LiaY and Cls1 as further evidence of possible interactions and that they may function concomitantly to mediate DAP-R.

We used fluorescence microscopy to determine whether Cls1 and LiaY colocalize with each other and within APL microdomains either at the septum or away from the septum in DAP-S (OG1RF/OG117) and DAP-R *Efs* strains, respectively. We generated a *liaY*-mCherry construct in pMSP3535 and were able to identify foci of LiaY proteins localized mostly at the mid-cell in DAP-S *Efs* OG1RFΔ*liaY* (Figure 6, C and E, and Supplemental Figure 15). In contrast, LiaY was visualized away from the mid-cell in DAP-R *Efs* OG117Δ*liaX*. Most importantly, we were able to show that, similar to Cls1, LiaY colocalized to APL microdomains in both DAP-S *Efs* OG1RFΔ*liaY* and DAP-R *Efs* OG117Δ*liaX* (Figure 6C and Supplemental Figure 15). Of note, *Efs* OG117Δ*liaX* expressed both the native *liaY* and *liaY-mCherry* due to the difficulties of generating a *liaY* deletion mutant in DAP-R backgrounds.

Lastly, we evaluated the in vivo colocalization of Cls1 and LiaY using a GFP-tagged Cls1 introduced into the chromosome, concomitant with *liaY*-*mCherry* expressed on pMSP3535. Figure 6, D and F, and Supplemental Figure 15 show that Cls1 and LiaY colocalize at the septum in DAP-S *Efs* OG117 and at nonseptal sites in DAP-R *Efs* OG117Δ*liaX*. Controls for potential self-aggregation of fluorescent tags were included to rule out artifactual localization patterns using pMSP3535 expressing GFP or mCherry alone, showing only a diffuse pattern with no discrete foci in either *Efs* OG117 (DAP-S) or OG117Δ*liaX* (DAP-R) (Supplemental Figure 16). Furthermore, quantification of colocalization between LiaY and NAO or LiaY and Cls1 showed much stronger association in DAP-R over DAP-S strains (Figure 6, C and D). The colocalization of these 2 proteins with the APL microdomains and each other, while not confirming direct interaction, does support our hypothesis that LiaY and Cls1 likely both cluster in the same membrane areas and are necessary to mediate DAP-R when LiaFSR is activated.

## Discussion

DAP is thought to target fluid areas of the membrane rich in PG (1) with eventual CM penetration likely causing inhibition of cell wall synthesis (2) and compromised cell envelope integrity (1). Enterococci counteract these negative effects by triggering a membrane adaptive response coordinated by members of the LiaFSR system (4, 5, 7, 8) to relocalize APL microdomains (6, 31), ultimately diverting DAP from the septum (4, 6). However, the mechanistic basis of this response is unknown. Here, we identified the major elements involved in this response and provide a mechanistic model to gain further understanding on how multidrug-resistant enterococci adapt their CMs to survive (32, 33).

**Figure 7:**
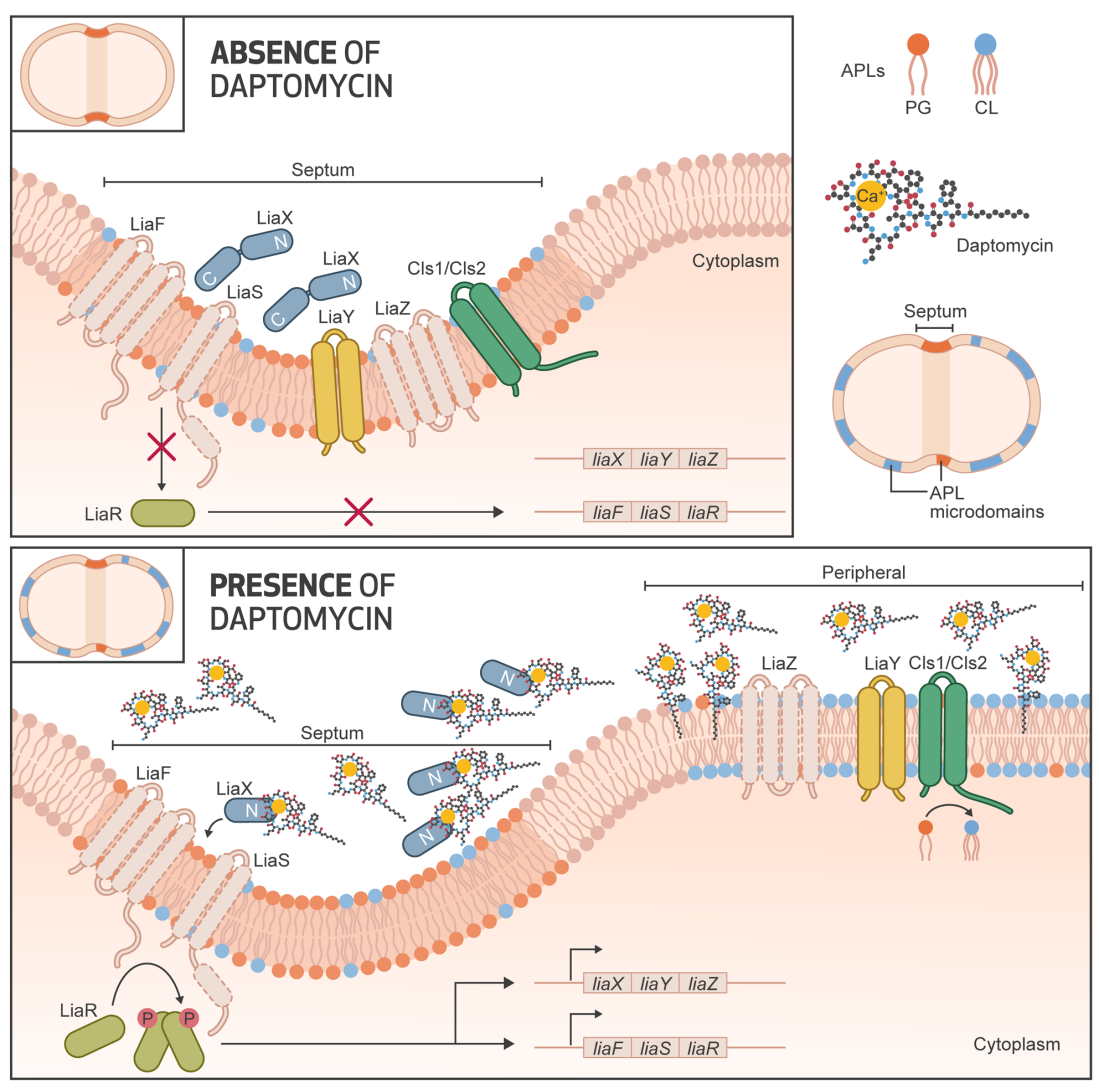
Mechanistic model of cell membrane adaptation in daptomycin-resistant *E. faecalis*. In the absence of daptomycin (top panel), anionic phospholipid microdomains are located at the bacterial division septum where other critical membrane-associated proteins involved in cell division, cell wall or lipid biosynthesis (such as *Cls1*) are also found. Members of the LiaFSR system (namely *liaXYZ*) are produced at basal levels and localize in septal areas. *LiaX* plays an inhibitory role through its C-terminal domain likely via interactions with members of the LiaFSR system. Upon activation of LiaFSR in the presence of daptomycin (bottom panel), the N-terminal domain of LiaX is released to the milieu and interacts with DAP. Concomitant changes in the C-terminus of LiaX cause “activation” of LiaY, generating interactions with Cls1. The LiaY-Cls1 complexes localize in areas away from the septum, presumably in regions where the membrane is being damaged by the antibiotic. Cls1 then produces high amounts of cardiolipin in these nonseptal areas, attracting DAP molecules to these membrane regions, resulting in alteration of the interaction of DAP with its transmembrane target (likely lipid II intermediates), preventing further DAP-mediated damage.

We first characterized the role of CL and Cls, which have frequently been associated with DAP-R without a clear mechanism (5, 8, 9). Our findings support a model in which Cls is upregulated upon development of DAP-R, acting as a bridge between activation of the LiaFSR system and DAP-R–associated changes in the CM. Interestingly, Cls1 is not regulated by LiaR (4). Nonetheless, previous work (34) has shown that exposure to DAP causes changes in expression of various genes including those involved in CM biogenesis, and we speculate that changes in phospholipid metabolism upon oligomerization of DAP in the CM could trigger the increased expression of *cls1* and *cls2*. In any scenario, CL-rich, nonseptal APL microdomains are likely to alter the CM oligomerization and translocation properties of DAP, as some previous published work suggests (14), and “trap” DAP in areas away from the division septum to protect septal cell division and peptidoglycan synthesis (6) (Figure 7).

*Efs* possesses more than 1 copy of the *cls* gene, and our results show that both Cls1 and Cls2 have overlapping functions. Although this functional redundancy is not surprising, the reasons for the preference for Cls1 during development of DAP-R are obscure, as are any differential roles between the 2 enzymes. Of note, overexpression of *cls2* appears to have deleterious effects on cell division (Supplemental Figure 5A), suggesting that Cls2 may have unique roles in certain biological contexts. Also, our lipidomics studies show a difference in levels of certain CL species synthesized by Cls1 or Cls2 (Table 3). These findings suggest that PG units containing particular fatty acids can be preferentially incorporated by individual Cls enzymes. Fatty acid tail length may allow CL species to alter the physicochemical properties of the CM, with previous studies showing that areas containing increased cyclic or decreased unsaturated fatty acids are likely less prone to the deleterious effects of DAP (24). Our results also provide further insights into the controversy surrounding the use of NAO staining. Historically, NAO has been proposed to bind CM regions that are rich in CL (35). However, it has been shown that NAO binds other APLs, such as PG or phosphatidic acid, in conditions of low (or no) CL content (36, 37), and we also show positive NAO staining in the absence of detectable levels of CL.

Another major finding of this work is the discovery of the function of LiaY, a protein that bridges the LiaFSR system with phospholipid homeostasis likely via colocalization with Cls. Interestingly, we could not generate a *liaY* mutant in DAP-R derivatives such as OG117Δ*liaX*, where the LiaFSR system is constitutively activated (4). In DAP-R OG117Δ*liaX*, attempts to delete *liaY* consistently resulted in a concomitant mutation in *liaFSR* that, presumably, shuts off the LiaFSR response, and the strain became hypersusceptible to DAP (Supplemental Table 3). The *liaY* gene is located in a cluster of 3 genes (*liaXYZ*), previously shown to be regulated by LiaR (4). Of note, *liaY* and the start codon of *liaZ* overlap, suggesting that they are cotranscribed. The inability to delete *liaY* in the DAP-R strain suggests that, under conditions of envelope stress, a stoichiometric balance between LiaY and LiaZ may influence cell viability in the absence of compensatory mutations. Supporting a deleterious role of unregulated *liaZ*, plasmid expression of *liaZ* in *Efs* OG1RF liaX*289Δ*liaYZ* led to an abnormal “clumping” phenotype (Supplemental Figure 14). Therefore, it is tempting to speculate that LiaY may modulate LiaZ function. Indeed, our studies show that LiaY and LiaZ may interact (Supplemental Figure 17). Nonetheless, our results indicate that both LiaY and Cls are both necessary and essential for the formation of nonseptal APL microdomains.

Finally, we propose a mechanistic model of CM activation triggered by DAP that leads to resistance (Figure 7). In DAP-S strains, CL and PG-rich APL microdomains are located at the bacterial division septum, favoring optimal functioning of critical proteins involved in vital cell processes, including cell division (10, 11) at the septum. In the absence of DAP, the LiaFSR system (and LiaXYZ) are produced at basal levels and localize in septal areas. Upon activation of LiaFSR, the increased expression of *liaXYZ* results in (a) changes in LiaX (4) that result in the upregulation of *liaY* and *cls1*, (b) LiaY and Cls1 relocalizing away from the septum, and (c) Cls producing CL in these nonseptal areas, preventing further DAP-mediated damage by altering the interaction of DAP with its septal targets. Cls2 is likely to play a similar role in this process to Cls1. Despite no evidence of direct interaction with LiaY, localization studies (Supplemental Figure 9) suggest that Cls2 may also relocalize away from the septum in DAP-R. Moreover, the APL microdomains are likely to serve as a signal for aberrant cell division and attract proteins involved in the divisome (as it has been previously shown; refs. 10, 11), preventing damage of cell division processes at the septum.

Overall, our results provide evidence to bridge the activation of the LiaFSR system with CL homeostasis as an adaptive response at the membrane level leading to DAP-R and provide further insights into the strategies that Gram^+^ bacteria deploy to avoid the killing effect of cell envelope–targeting antibiotics.

## Methods

### Sex as a biological factor

Sex was not considered as a biological factor in this study.

### Bacterial growth

*E. coli* strains were cultured at 37°C with agitation either in broth or on agar plates with Luria-Bertani (Becton Dickinson) with appropriate antibiotics (Sigma-Aldrich) added for maintaining plasmids if needed: 300 μg/mL erythromycin for pMSP3535 (20), 25 μg/mL gentamicin for pHOU1 (29, 30)/pAT392 (21), 50 μg/mL kanamycin or 100 μg/mL ampicillin for bacterial 2-hybrid plasmids (see below), 15 μg/mL chloramphenicol for pCE (18). Enterococcal strains were cultured at 37°C with agitation either in broth or on agar plates with Brain Heart Infusion or Tryptic Soy Broth (Becton Dickinson) with appropriate antibiotics (Sigma-Aldrich) added for maintaining plasmids if needed: 15 μg/mL erythromycin for pMSP3535, 150 μg/mL gentamicin for pHOU1/pAT392, 15 μg/mL chloramphenicol for pCE. *Efs* OG1RF can be selected with fusidic acid (25 μg/mL), and *Efs* CK111 can be selected with spectinomycin (1,000 μg/mL).

### Bacterial mutagenesis and complementation

Sequences of the primers used for mutagenesis, screening, and other plasmid construction are located in Supplemental Table 2 (Sigma-Aldrich). Bacterial strains and plasmids used or generated in this study are described in Supplemental Table 1.

Mutagenesis with the PheS counterselection system (29, 30) was used to generate deletion mutants in the *Efs* OG1RF background strain. Briefly, upstream and downstream fragments (approximately 1,000 bp each) of the gene to be deleted were amplified with Phusion (New England Biolabs) according to manufacturer instruction. Fragments were joined via crossover PCR using PrimeStar Max (Takara Bio) per manufacturer instruction. Crossover fragment ends and vector pHOU1 were digested with either BamHI, EcoRI, or XbaI (New England Biolabs) at 37°C for 2 hours. T4 DNA ligase (New England Biolabs) was used to ligate the crossover fragment into pHOU1 with overnight incubation at 16°C. pHOU1 containing the flanking regions was transformed into *E*. *coli* EC1000, electroporated in *Efs* CK111, and conjugated into *Efs* OG1RF. After confirming the first recombination event after conjugation into *Efs* OG1RF, colonies were streaked onto minimal media agar containing p-chloro-Phe to release the plasmid. Deletion of the gene of interest were confirmed via PCR and then by Sanger sequencing (Azenta) and/or whole genome sequencing.

Deletion mutants and chromosomal protein tags in *Efs* OG117 were generated using the CRISPR-Cas9 mutagenesis system (18). For the deletion mutants, upstream and downstream flanking regions (approximately 1,000 bp) were amplified using PrimeStar HS (Takara Bio) and cloned into the vector pCE along with a 30 bp site-specific spacer sequence (in the gene to be deleted) using Gibson assembly (Gibson Assembly Cloning Kit, New England Biolabs) using 1 μg of fragments and plasmid at a 3:1 ratio then incubating at 50°C for 4 hours. A total of 2 μL of the Gibson assembly was then transformed into NEB 5-alpha Competent *E*. *coli* (New England Biolabs) according to manufacturer protocol and plated onto selective LB agar containing 15 μg/mL chloramphenicol. Positive colonies were confirmed with Sanger sequencing (Azenta) and electroporated into *Efs* OG117 or its derivatives. Colonies successfully mutagenized were plated on minimal media agar containing p-chloro-Phe to eject the plasmid. Colonies containing the deletion were confirmed via Sanger sequencing (Azenta) and/or whole genome sequencing. For protein tagging, the gene encoding the fluorescent protein of interest was cloned upstream or downstream of the gene of interest with a glycine-glycine-serine linker to generate the fragment for Gibson assembly.

Complementation was carried out in trans from either the nisin inducible vector pMSP3535 or the constitutively expressing vector pAT392. The gene of interest was amplified by PCR with Phusion (New England Biolabs), digested with BamHI and XbaI (New England Biolabs), and ligated into the appropriate vector with T4 DNA ligase (New England Biolabs). Recombinant plasmids were confirmed by PCR (GoTaq DNA Polymerase, Promega) and Sanger sequencing (Azenta), prior to electroporation into the strain of interest. Empty vectors (for fluorescence studies, vector containing the fluorescent protein alone) were also electroporated into the strains of interest as a control.

### MIC

MICs for DAP were determined using Etest (Biomerieux). Strains were diluted to make a 0.5 McFarland standard that was then inoculated on BHI agar (Becton Dickinson) containing appropriate antibiotics (Sigma-Aldrich) for selection of plasmids as described above. Etest strips were placed on the plate and incubated at 37°C, and MIC results were interpreted after 24 hours.

### qPCR

Oligonucleotides used for expression studies are described in Supplemental Table 2. Cell cultures were grown to either exponential or stationary phase in tryptic soy broth at 37°C and pelleted. RNA was extracted using the PureLink RNA Extraction Kit (Invitrogen), and contaminating DNA was removed with TurboDNase (Invitrogen). cDNA was generated using SuperScriptII (Thermo Fisher Scientific) reverse transcriptase according to manufacturer instructions. A total of 5 ng of cDNA was loaded in triplicate and qPCR was used to evaluate gene expression using SsoAdvanced Universal SYBR Green Supermix (Bio-Rad) in the CFX96 Touch Real-Time PCR Detection System (Bio-Rad) as follows: 30 seconds at 95°C, 35 cycles; 15 seconds at 95°C; 30 seconds at 60°C. Relative gene expression was calculated using the Pffafl (19) method with the gene encoding GyrB or 16S rRNA as the reference for normalization. The LinReg program was used to determine primer efficiency. A 2-tailed *t* test was used to evaluate differential gene expression between *cls1* and *cls2* for each growth condition.

### Lipid extraction and liquid chromatography–MS (LC-MS)

#### Reagents.

High-performance LC grade solvents (water, acetonitrile, chloroform, and methanol) and ammonium acetate (Optima LC/MS grade) were purchased from Thermo Fisher Scientific. All PC and PE lipid standards used for collisional cross section (CCS) calibration were purchased from Avanti Polar Lipids (38).

#### Lipid extraction.

Lipid extraction was conducted based on the Bligh and Dyer method as described elsewhere (39–41). Briefly, bacteria broth was collected after cultivation in 24 hours, rinsed with 1× PBS, spun, and dried with a speed-vac (Thermo Fisher Savant). A total of 1 mL of water was then added to the pelleted and dried bacteria. The resulting suspensions were sonicated in an ice bath for 30 minutes to dislodge the dried pellets and homogenize the suspension. In total, 4 mL of chilled solution of chloroform and methanol (1:2 v/v) was added to each tube, followed by 5 minutes of vigorous vortex and the addition of 1 mL of chilled chloroform and 1 mL of chilled water. The samples were then rigorously vortexed for 1 minute and centrifuged for 10 minutes at 4°C and 2,000*g* to separate the organic and aqueous layers. The organic layers were collected to new 10 mL glass centrifuge tubes (Thermo Fisher Scientific) and dried in a vacuum concentrator. The dried lipid extracts were reconstituted with 500 μL of 1:1 chloroform/methanol and stored in –80°C. For HILIC-IM-MS analysis, 5 μL of lipid extract was transferred into LC vials, dried under nitrogen, and redissolved in 100 μL of 2:1 acetonitrile/methanol.

#### LC.

Bacterial lipids were separated by a Waters UPLC (Waters Corp.) as described previously (22, 23). Briefly, HILIC was performed with a Phenomenex Kinetex HILIC column (2.1 × 100 mm, 1.7 μm) maintained at 40°C at a flow rate of 0.5 mL/min. The solvent system consisted of: (a) 50% acetonitrile/50% water with 5 mM ammonium acetate, and (b) 95% acetonitrile/5% water with 5 mM ammonium acetate. The gradient program was optimized as follows: 0–1 minute, 100% B; 4 minutes, 90% B; 7–8 minutes, 70% B; and 9–12 minutes, 100% B. A sample injection volume of 5 μL was used for all analyses.

#### IM-MS.

The Waters Synapt G2-XS platform was used for lipidomics analysis. Effluent from the UPLC was introduced through the electrospray ionization (ESI) source. ESI capillary voltages of +2.0 and –2.0 kV were used for positive and negative analyses, respectively. Additional ESI conditions were as follows: sampling cone, 40 V; extraction cone, 80 V; source temperature, 150°C; desolvation temperature, 500°C; cone gas, 10 L/hour; and desolvation gas, 1,000 L/hour. Mass calibration over *m/z* 50–1200 was performed with sodium formate. Calibration of IM measurements was performed as previously described (42). IM separation was performed with a traveling wave height of 40 V and velocity of 500 m/s. Data was acquired for *m/z* 50–1,200 with a 1-second scan time. Untargeted MS/MS (MS^E^) was performed in the transfer region with a collision energy ramp of 35–45 eV. Mass and drift time correction was performed after acquisition using the leucine enkephalin lockspray signal.

#### Data analysis.

Data alignment, chromatographic peaks detection, and normalization were performed in Progenesis QI (Nonlinear Dynamics). A pooled quality control sample was used as the alignment reference. The default “All Compounds” method of normalization was used to correct for variation in the total ion current amongst samples. Lipid identifications were made based on *m/z* (within 10 ppm mass accuracy), retention time and CCS with an in-house version of LipidPioneer, modified to contain the major lipid species observed in *E*. *faecalis*, including DGs, DGDGs, PGs, CLs, and LysylPGs (LPGs) with fatty acyl compositions ranging from 25:0 to 38:0 (total carbons/total degree unsaturation), and LiPydomics (22, 43, 44).

Results were reported normalized to the 15:0/15:0 phosphatidylethanolamine (Avanti Polar Lipids) control for overall lipid classes by abundance or percentage of individual lipid species to the whole lipid class. A *t* test was used to compare *E*. *faecalis* OG117 with OG117Δ*liaX*. Two-way ANOVA was used to compare *E*. *faecalis* OG117 and the individual *cls* deletions (or *Efs* OG117Δ*liaX* and the individual *cls* deletions), and a *t* test was used to compare the individual *cls* deletions with each other.

### Fluorescence microscopy

Images were adjusted for “Black Balance” per BZ-X800 Image Analysis Software with individual representative image selected for figures. All adjustments were made to whole images.

#### APL microdomains/NAO staining.

Cells were grown in tryptic soy broth, and 1 μM NAO was added in early exponential phase. Cells were grown in the dark at 37°C with agitation to early stationary phase prior to washing with phosphate buffered saline and adherence to poly-L-lysine coverslips. Coverslips were mounted to glass slides with ProGlass media and visualized using a Keyence BZ-X800 fluorescence microscope with filters for GFP (excitation: 470 nm/emission: 525 nm). Quantification of at least 50 cells per strain in triplicate was done to evaluate whether regions of fluorescence intensity were septally located. One-way ANOVA was used to evaluate the differences between the strains.

#### Fluorescent tags.

Proteins of interest were tagged with either GFP or mCherry. For fluorescently tagged proteins expressed in *trans*, growth in brain heart infusion (BHI) media was supplemented with appropriate antibiotics (gentamicin 150 μg/mL or erythromycin 15 μg/mL) for selection and/or induction (75–150 ng/mL nisin added after 1 hour of growth continuing to early stationary phase). If NAO staining was concurrent, 1 μM NAO was added in early exponential phase. Cells were grown to late stationary phase (optimal stage for concurrent expression of both fluorescently tagged proteins for visualization) in the dark at 37°C with agitation; they were washed and mounted to coverslips as described above. Visualization was performed using a Keyence BZ-X800 fluorescence microscope with filters for GFP (excitation: 470 nm/emission: 525 nm) or Texas Red (excitation: 560 nm/emission: 630 nm). Overlay images were produced using the BZ-X800 Analyzer software (Keyence). Representative images of protein localization/colocalization are shown.

#### Fluorescence quantification.

Quantification and colocalization of fluorescence for LiaY-mCherry, NAO staining, and Cls1-ReAsH was performed using the Coloc2 plugin in ImageJ (NIH; https://imagej.net/ij/index.html). The Pearson Correlation Coefficient was calculated for 14–16 ROIs for each condition. The Fisher Transformation was used to calculate *Z* scores from the Pearson Correlations and determine the mean *Z* score for each condition. The Fisher Inverse Transformation was used to calculate the Pearson Correlation Coefficient from the mean *Z* score. This coefficient was used to calculate the 95% CI based on the number of ROIs measured for each condition.

#### Tetracysteine-based fluorescence.

Proteins of interest were tagged with a tetracysteine motif (26) at the N-terminal domain and expressed in pMSP3535 in *E*. *faecalis* using erythromycin (15 μg/mL) for selection of plasmid containing clones. Cultures were grown in tryptic soy broth at 37°C for 2.5 hours prior to addition of 50 ng/mL of nisin for induction. Cultures were continued for another 3.5 hours prior to addition of 1 μM NAO and/or 1 μM ReAsH-EDT2 reagent (Invitrogen). Cultures were incubated for 1 hour at room temperature and washed with 1× BAL wash buffer per manufacturer kit instructions (Invitrogen TC-ReAsH II In-Cell Tetracysteine Tag Detection Kit [Red Fluorescence], for live-cell imaging). Cultures were adhered to coverslips and mounted to glass slides for visualization with a Keyence BZ-X800 with filters for GFP (excitation: 470 nm/emission: 525 nm) or Texas Red (excitation: 560 nm/emission: 630 nm). Representative images of protein localization/colocalization are shown.

#### HADA and NAO colocalization.

Cells were grown to exponential phase in tryptic soy broth followed by addition of NAO at 1 μM and HADA at 0.5 mM. Cultures were adhered to coverslips and mounted to glass slides for visualization with a Keyence BZ-X800 with filters for GFP (excitation: 470 nm/emission: 525 nm) or DAPI (excitation: 360 nm/emission: 460 nm). Representative images of protein localization/colocalization are shown.

### Bacterial 2-hybrid system

Oligonucleotides used for bacterial 2-hybrid studies are shown in Supplemental Table 2. Protein-to-protein interactions were evaluated using the Bacterial Two-Hybrid System (Euromedex) according to the manufacturer instructions. Briefly, proteins of interest were tagged with catalytic adenylate cyclase domains (T18 or T25) at their N- or C-terminal domains and coexpressed in *E*. *coli* BTH101 (a reporter strain lacking adenylate cyclase) in Luria Bertani broth containing 50 μg/mL kanamycin, 100 μg/mL ampicillin, and 1 μM Isopropyl β-D-1-thiogalactopyranoside (IPTG) as the inducer. The strength of the interaction was determined by a β-galactosidase assay (per Euromedex instructions) after incubation at 30°C for 48–72 hours. Briefly, induced cells were permeabilized with toluene (Sigma-Aldrich) and SDS (Thermo Fisher Scientific) prior to addition of 0.4% o-nitrophenol-β-galactoside (Sigma-Aldrich). Solutions were incubated at 30°C to allow for the colorimetric reaction to proceed. Reaction was stopped upon development of visible yellow color with 1M Na_2_CO_3_ (Thermo Fisher Scientific)._._ The absorbance at OD_420_ nm was read using a BioTek Synergy H1 Microplate Reader. Activity units were reported normalized to cell density and reaction time.

### Reagents and resource

Oligonucleotides (Sigma-Aldrich) used in this study can be found in Supplemental Table 2.

### Materials availability

Requests for use of specific generated strains can be directed to the lead contact and may be subject to a material transfer agreement. Other materials are listed in Supplemental Table 4.

### Statistics

Statistical details for each study can be found in the figure legends. The analyses were performed in GraphPad Prism, with the analysis type and *n* (number of replicates or cells counted) indicated in the figure legend for each experiment. For analysis with 1- or 2-way ANOVA (specified in figure legends), corrections for multiple comparisons were performed. Statistical significance is defined in each figure legend. Data are shown as mean ± SEM.

### Study approval

Approval was not required, as the the study did not include patients or animal subjects.

### Data availability

Data reported in this paper will be shared by the lead contact upon request. Data for figures are included in the data table. Please contact the corresponding author by email listed above for any additional data requests. This paper does not report original code. Microbial strains and microbial source material for in vitro studies are reported in Supplemental Table 1. Values for all data points in graphs are reported in the Supporting Data Values file.

## Author contributions

AHN, TTT, DP, AK, HV, and CAA designed research. AHN, TTT, DP, VP, and RZ performed research. KH, LX, and RZ contributed new reagents/analytic tools. AHN, TTT, DP, KH, RZ, AK, WRM, EM, YS, LX, HV, and CAA analyzed data. AHN and CAA wrote the paper.

## Supplementary Material

Supplemental data

Supporting data values

## Figures and Tables

**Table 1:**
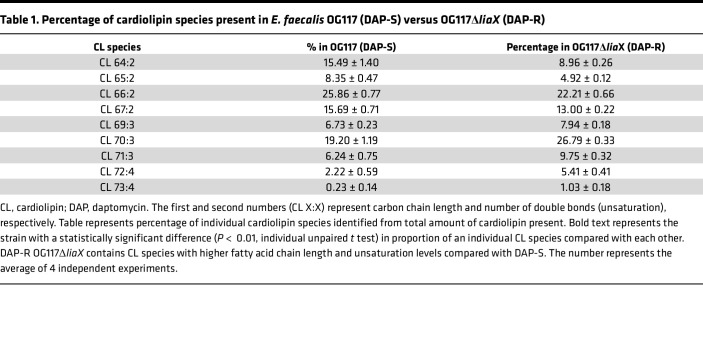
Percentage of cardiolipin species present in *E. faecalis* OG117 (DAP-S) versus OG117Δ*liaX* (DAP-R)

**Table 2:**
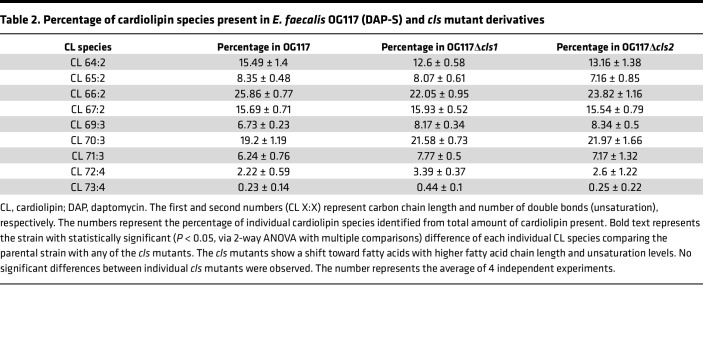
Percentage of cardiolipin species present in *E. faecalis* OG117 (DAP-S) and *cls* mutant derivatives

**Table 3:**
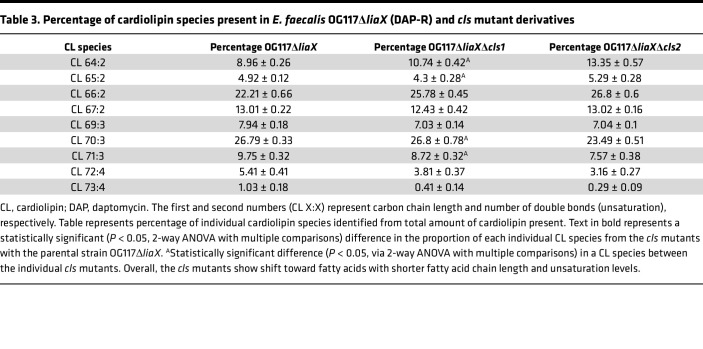
Percentage of cardiolipin species present in *E. faecalis* OG117Δ*liaX* (DAP-R) and *cls* mutant derivatives

## Version 1 10/15/2024

In-Press Preview

## Version 2 11/22/2024

Electronic publication
