# Supplementary material for: Molecular basis of cell membrane adaptation in daptomycin-resistant Enterococcus faecalis
Source: JCI Insight. 2024 Nov 22;9(22):e173836. doi: 10.1172/jci.insight.173836 (PMC11601895; doi:10.1172/jci.insight.173836)
Supplement: Supplemental data [file jciinsight-9-173836-s185.pdf]

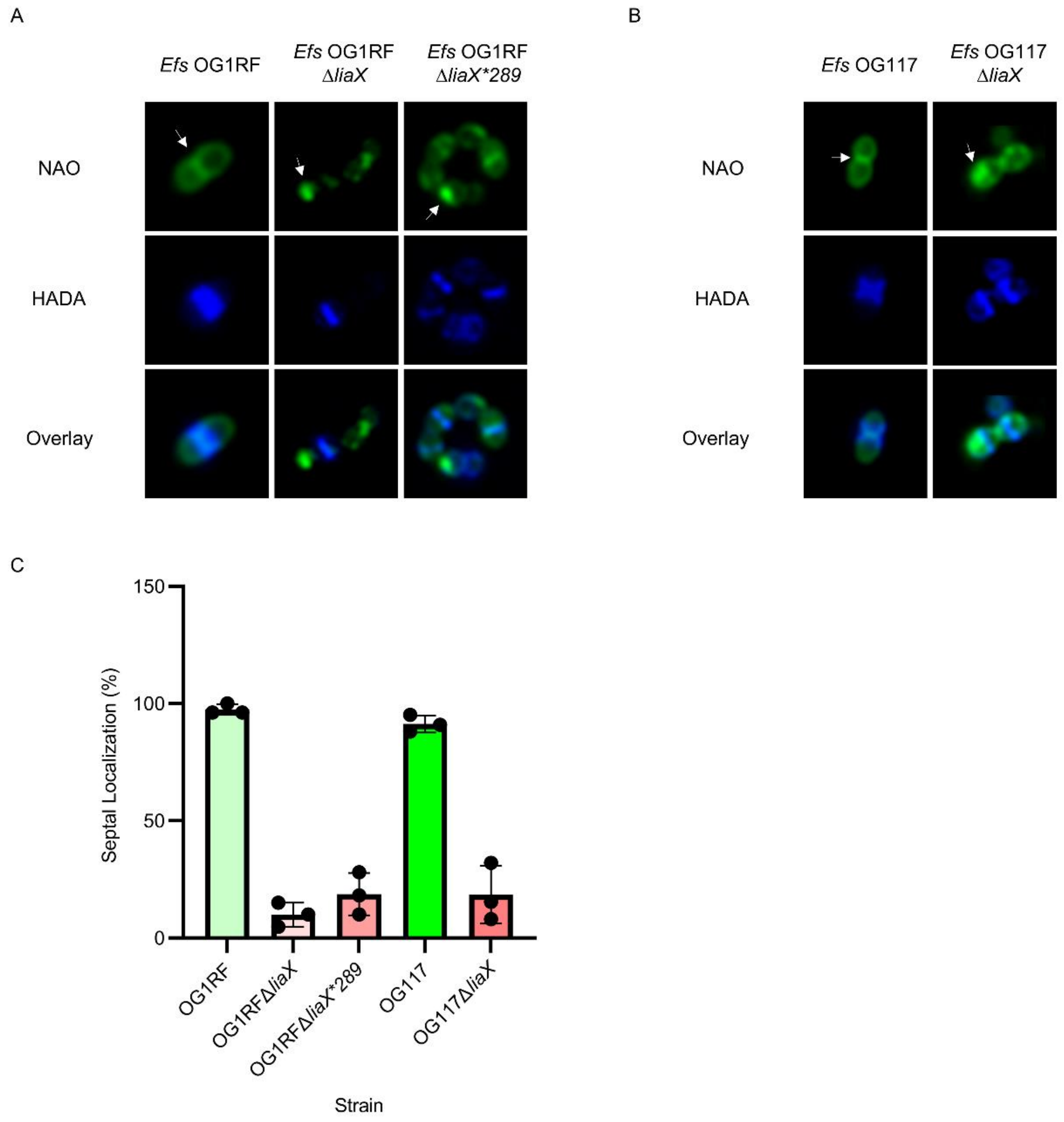

Supplementary Figure 1: NAO and HADA co-localization

A) NAO (top row), HADA (middle row) and overlay (bottom row) images of *Efs* OG1RF with or without deletion of *liaX* or *liaX**289. White arrows represent anionic phospholipid microdomains at mid-cell or non-mid-cell locations.

B) NAO (top row), HADA (middle row) and overlay (bottom row) images of *Efs* OG117 with or without deletion of *liaX*. White arrows represent anionic phospholipid microdomains at mid-cell or non-mid-cell locations.

C) Quantification of septal localization per strain, minimum >20 cells/strain, n=3.

Whole images were adjusted for “Black Balance” per BZ-X800 Image Analysis Software with individual representative selected.

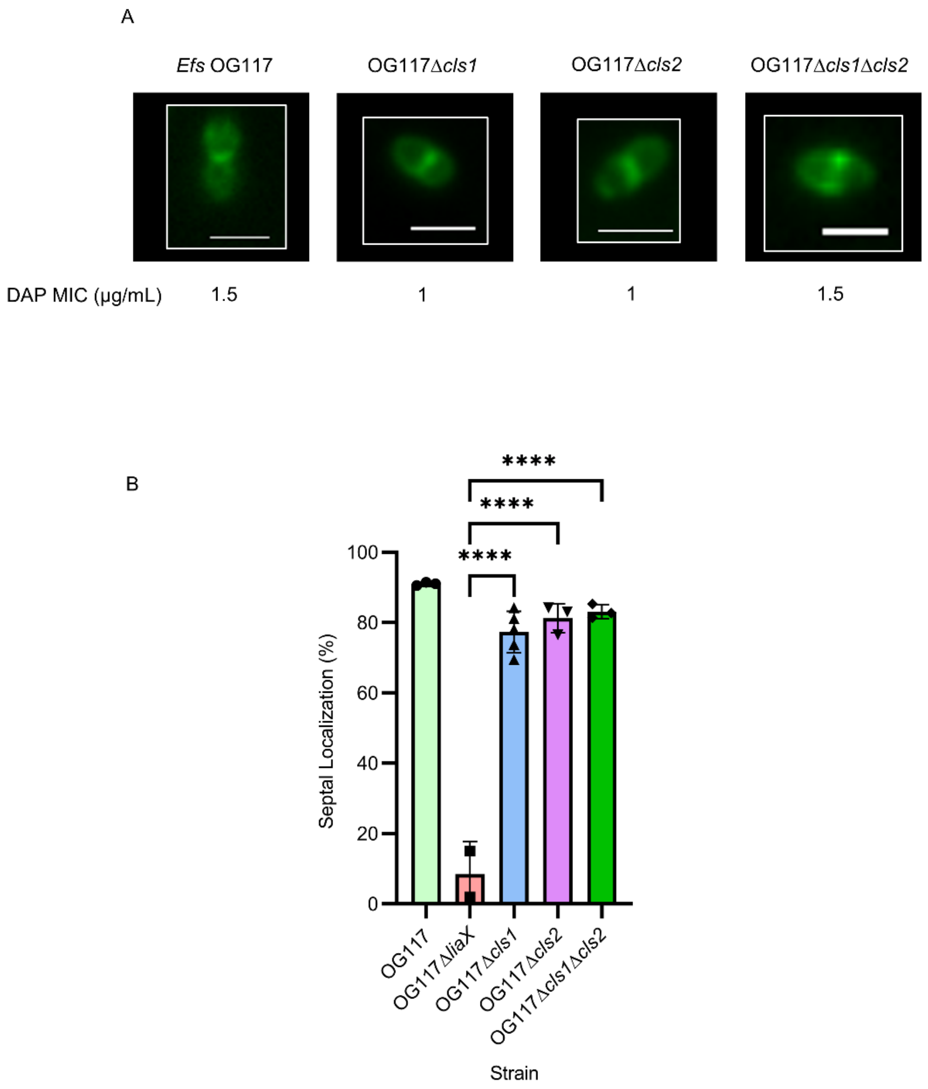

Supplementary Figure 2: Characterization of cls deletions in DAP-S Efs OG117.

A) Evaluation of anionic phospholipid localization using NAO staining in deletions of *cls1* and/or *cls2* in DAP-S *Efs* OG117 with DAP MIC (below image).

(B) Quantification of septal localization of anionic phospholipid microdomains with NAO in *cls* mutant derivatives of *Efs* OG117 by counting a minimum of 50 cells per replicate (n=3-6 replicates). **p<0.01, ***p<0.001, ****p<0.0001 via one-way ANOVA with multiple comparisons

Whole images were adjusted for "Black Balance" per BZ-X800 Image Analysis Software with individual representative selected.

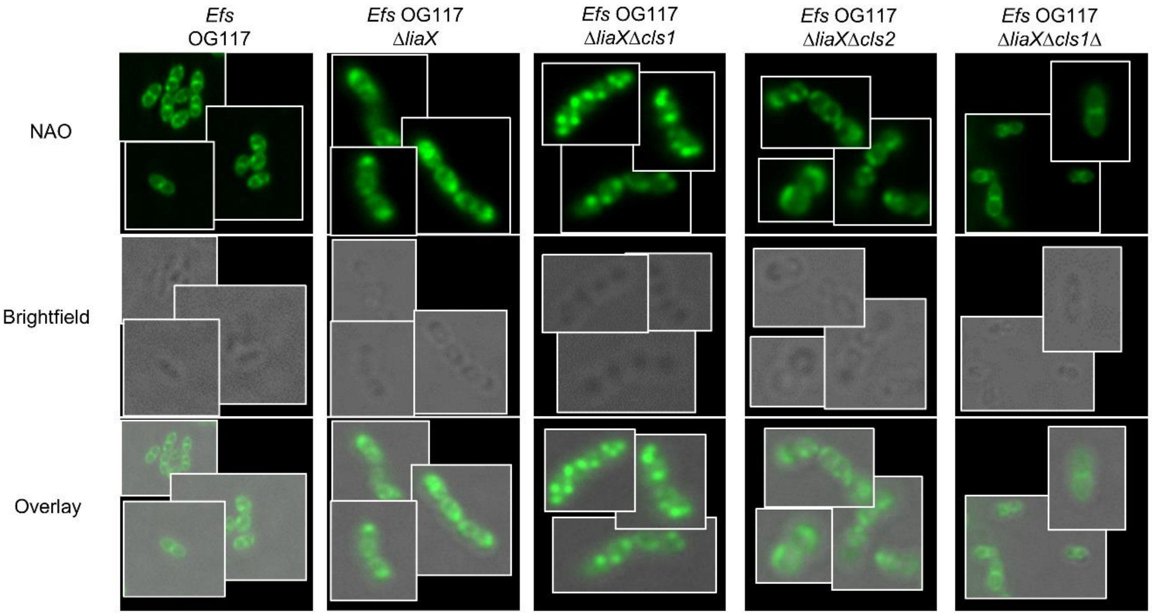

Supplementary Figure 3: Additional Images of cls mutants
Evaluation of anionic phospholipid domains using NAO staining (top row) in deletions of *cls* mutants with brightfield image (middle row) and overlay (bottom row). Whole images were adjusted for "Black Balance" per BZ-X800 Image Analysis Software with individual representative selected.

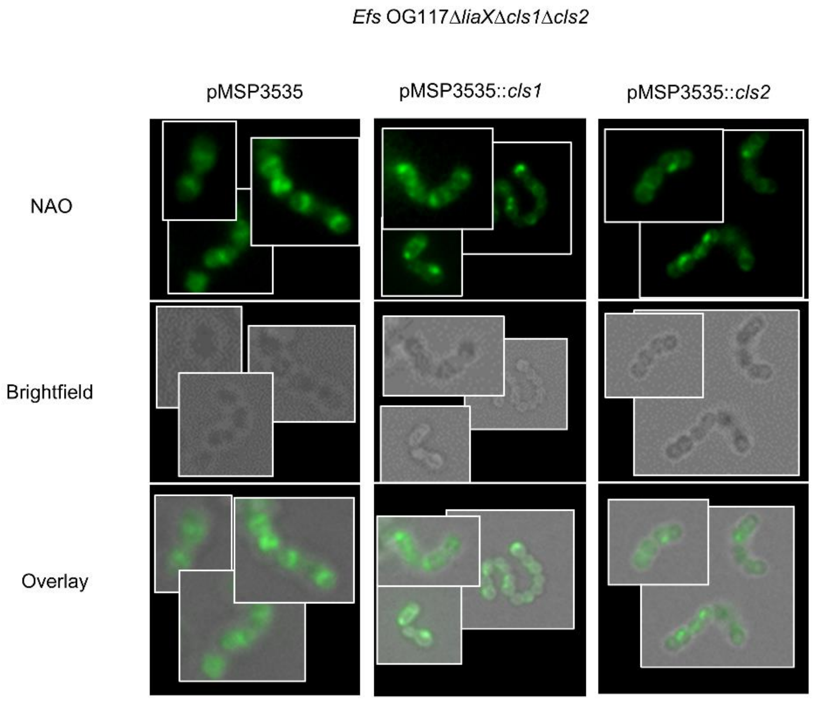

Supplementary Figure 4: Additional images of cls *mutants and trans-complementation*
Evaluation of anionic phospholipid domains using NAO staining (top row) in deletions of *cls* mutants complemented with either *cls1* or *cls2* in pMSP3535 with brightfield image (middle row) and overlay (bottom row). Whole images were adjusted for “Black Balance” per BZ-X800 Image Analysis Software with individual representative selected.

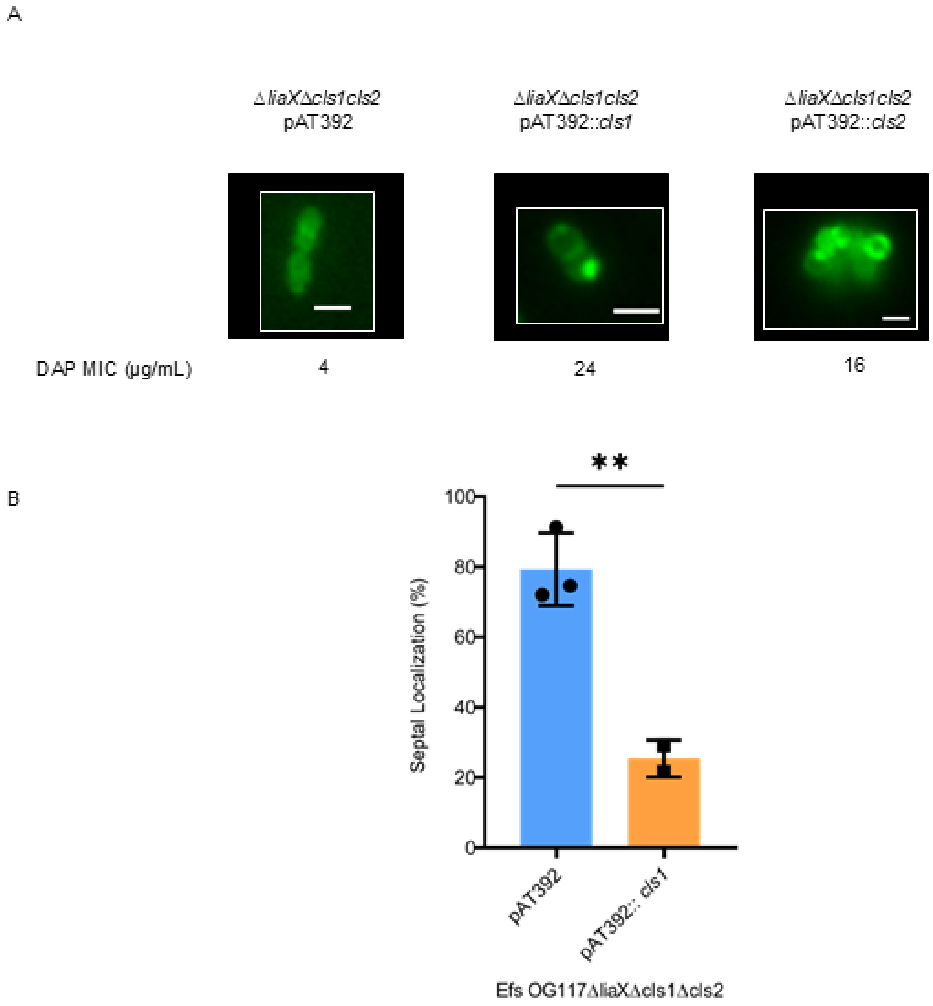

Supplementary Figure 5: Anionic phospholipid localization using NAO staining in DAP-R E. faecalis cls *mutant trans complementation*
(A) *E. faecalis* OG117Δ*liaX*Δ*cls1*Δ*cls2* transformed with pAT392 and derivatives containing *cls1* or *cls2* from. Scale bar (white) at 2µm
(B) Quantification of septal localization of anionic phospholipid microdomains with NAO staining in *E. faecalis* OG117Δ*liaX*Δ*cls1*Δ*cls2* transformed with pAT392 and derivatives containing *cls1*. *p<0.05; **p<0.001, n=2 via t-test
Whole images were adjusted for “Black Balance” per BZ-X800 Image Analysis Software with individual representative selected.

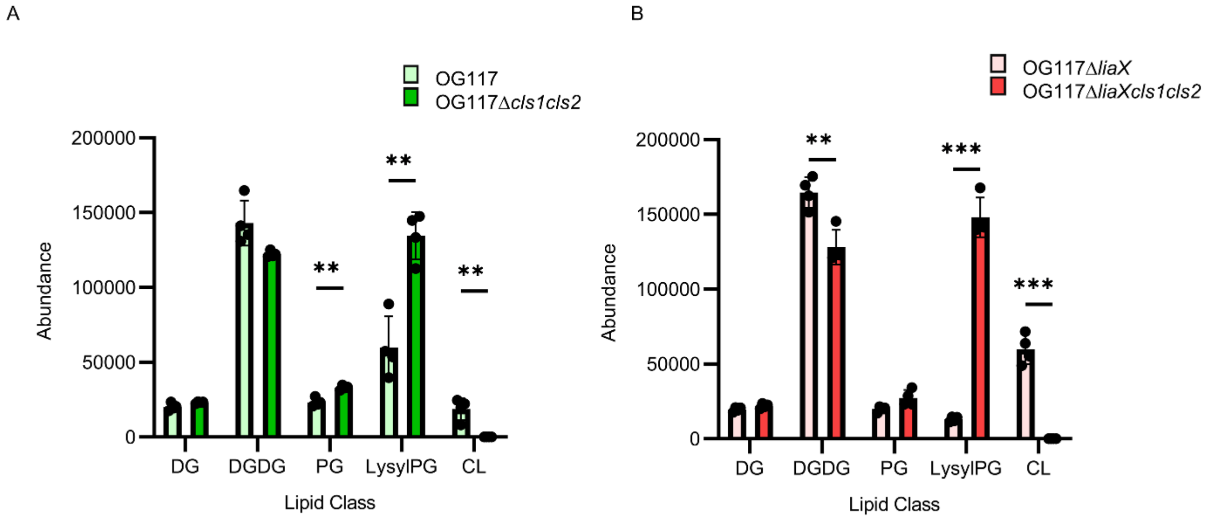

Supplementary Figure 6: Lipid Class Changes in cls *double deletion strains*

Quantification of cardiolipin content in in double *cls* deletions in DAP-S *E. faecalis* OG117 (A) and DAP-R *E. faecalis* OG117Δ*liaX* (B)
DG= diacylglycerol, DGDG = diglycodiacylglycerol, PG = phosphatidylglycerol, LysylPG = lysyl-phosphatidylglycerol, CL = cardiolipin
*p<0.05, n=4 via individual unpaired t-tests

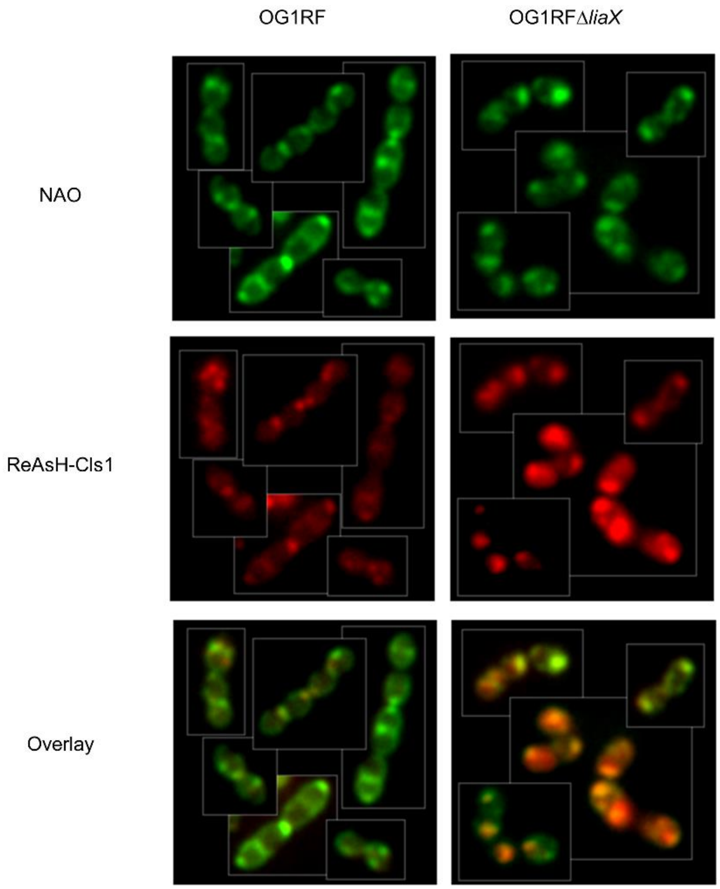

Supplementary Figure 7: Additional ReAsH-Cls1-NAO images
Representative images of NAO staining (top panel), tetracysteine-tagged Cls1 with ReAsH reagent (red fluorescence, middle panel) and overlay of both images (bottom panel) for strain listed with expression of *cls1-ReAsH* expressed from pMSP3535. Whole images were adjusted for “Black Balance” per BZ-X800 Image Analysis Software with individual representative selected.

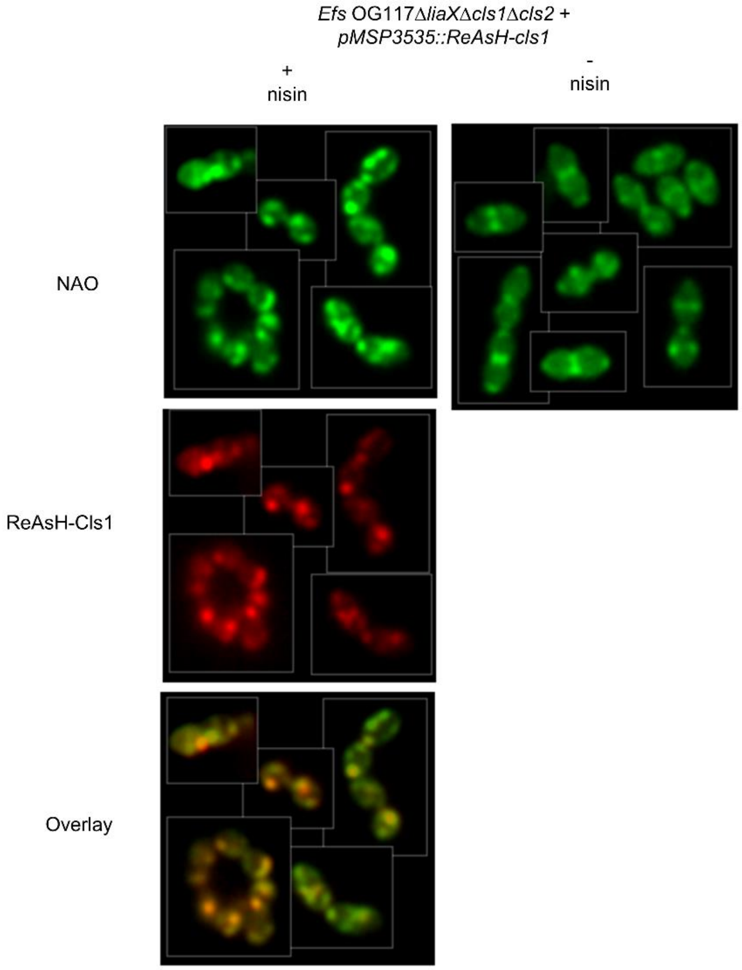

Supplementary Figure 8: Additional ReAsH-NAO pMSP3535 complementation images
Representative images of NAO staining (top panel), tetracysteine-tagged Cls1 with ReAsH reagent (red fluorescence, middle panel) and overlay of both images (bottom panel) for strain listed with expression of *cls1-ReAsH* expression from pMSP3535 with or without 50ng/mL nisin induction. Whole images were adjusted for “Black Balance” per BZ-X800 Image Analysis Software with individual representative selected.

pMSP3535::*gfp-cls2*

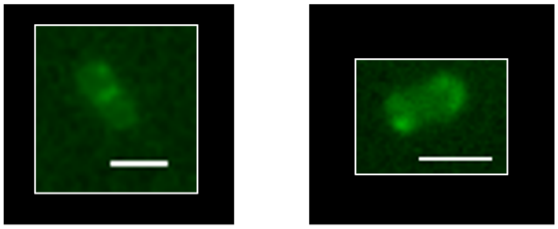

OG117Δ*cls1* OG117Δ*liaX*Δ*cls1*

Supplementary Figure 9. Cls2 re-localizes in the absence of Cls1 in DAP-S and DAP-R E. faecalis *strains.*
Cls2 was tagged with GFP and expressed on pMSP3535 in either DAP-S *Efs* OG117Δ*cls1* or DAP-R *Efs* OG117Δ*liaX*Δ*cls1.* Introduction of *gfp-cls2* highlight the septal and non-septal localization of Cls2 in DAP-S OG117Δ*cls1* and DAP-R OG117Δ*liaX*Δ*cls1*, respectively. Scale bar (white) at 2 µm.
Whole images were adjusted for “Black Balance” per BZ-X800 Image Analysis Software with individual representative selected.

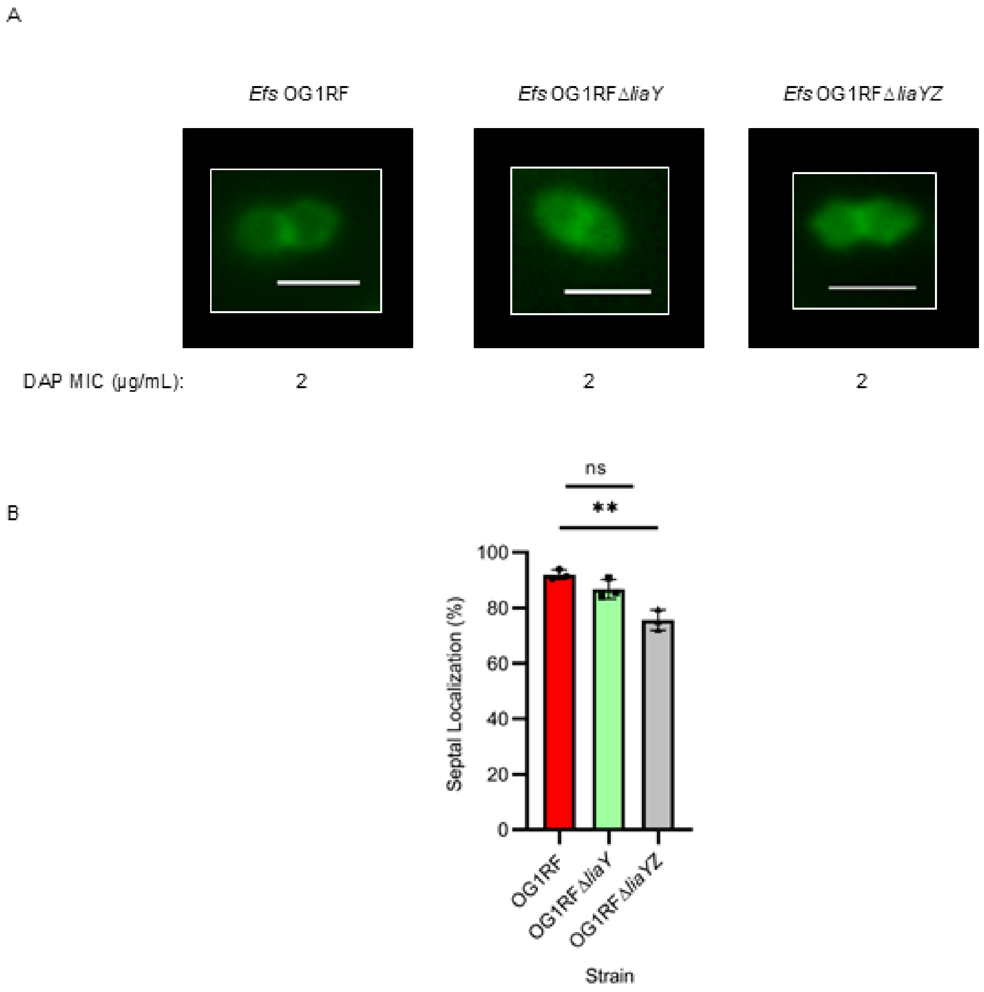

Supplementary Figure 10. Deletions of liaYZ *in DAP-S* E. faecalis *OG1RF in which the LiaFSR system is not activated.*
(A) NAO staining of anionic phospholipid microdomains staining with of *Efs* OG1RF, *Efs* OG1RFΔ*liaY*, and *Efs* OG1RFΔ*liaYZ* showing septal localization of microdomains without changes in DAP MIC. Scale bar (white) at 2 µm.
(B) Quantification of septal localization of anionic phospholipid microdomains with NAO staining in *cls* mutants. **p<0.001.via one way ANOVA with multiple comparisons
Whole images were adjusted for "Black Balance" per BZ-X800 Image Analysis Software with individual representative selected.

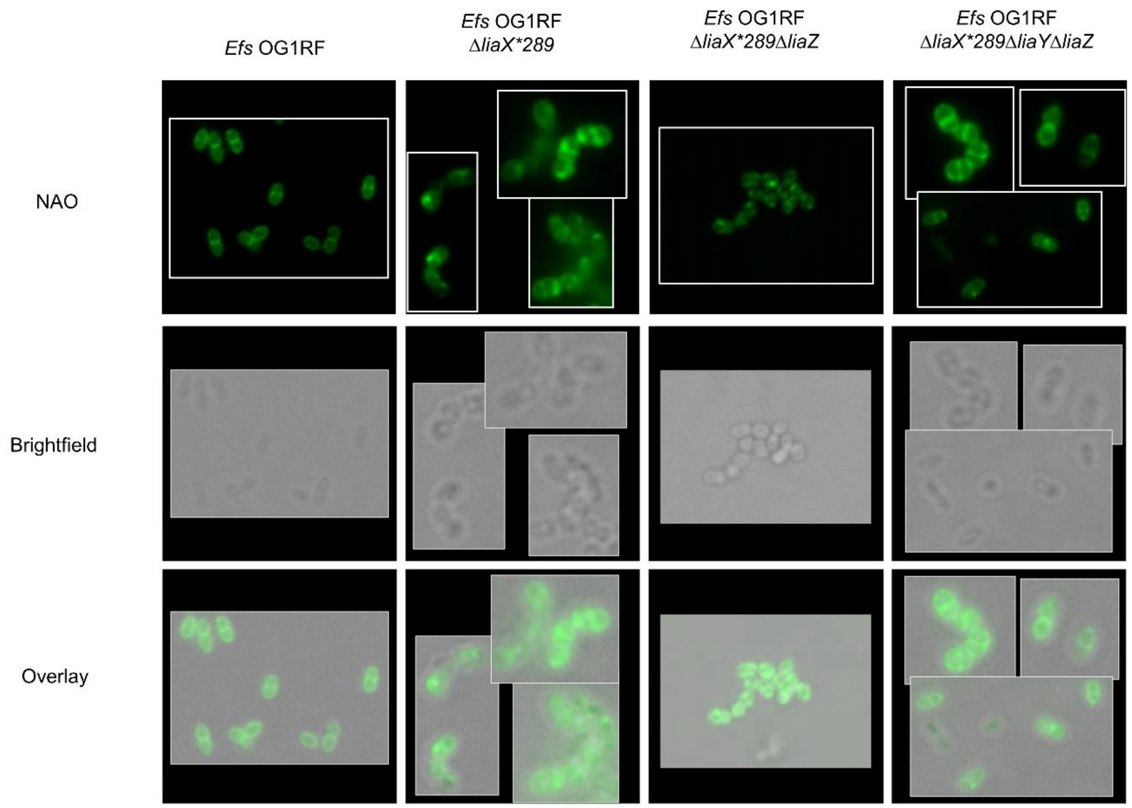

Supplementary Figure 11: Additional images of liaYZ *mutants*

Evaluation of anionic phospholipid domains using NAO staining (top row) in deletions of *liaYZ* mutants with brightfield image (middle row) and overlay (bottom row). Whole images were adjusted for “Black Balance” per BZ-X800 Image Analysis Software.

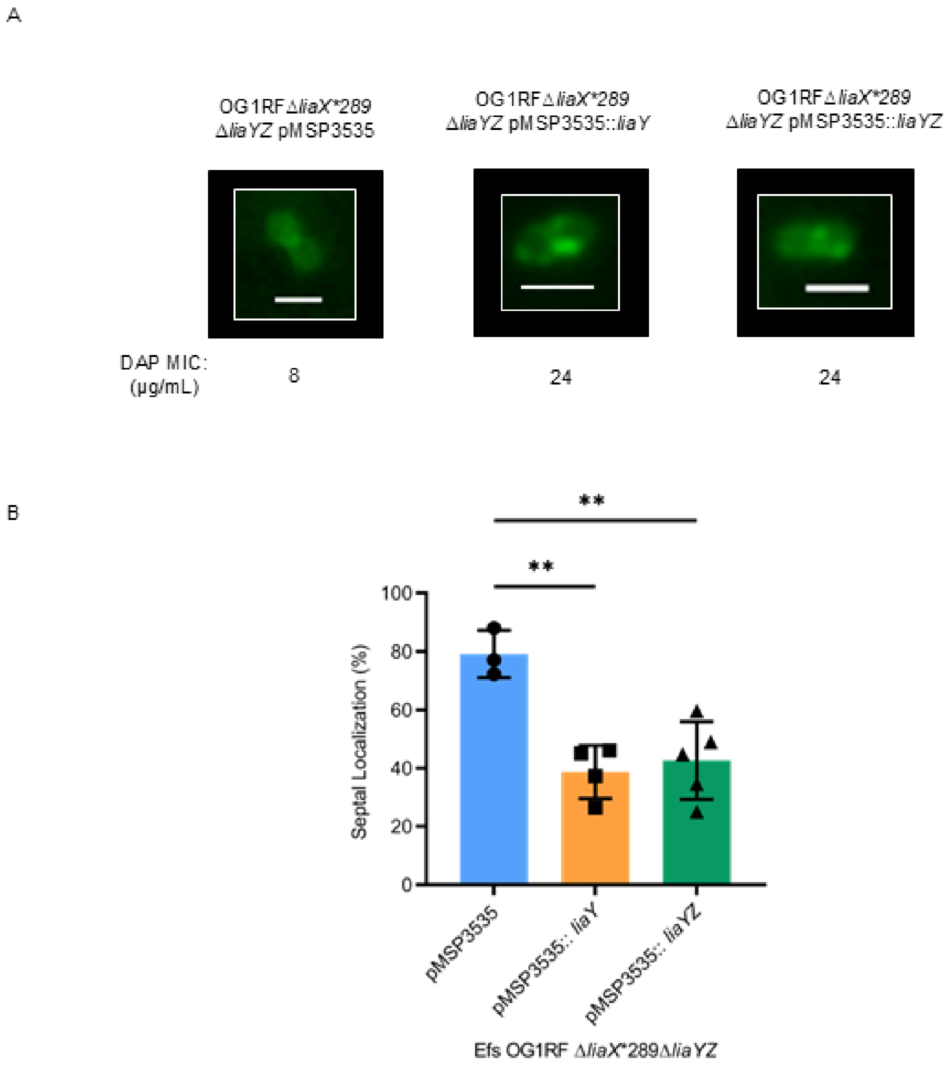

*Supplementary Figure 12: Anionic phospholipid localization using NAO staining in DAP-R E. faecalis OG1RFΔliaX*289ΔliaYZ and derivatives*

(A) *E. faecalis* OG1RFΔ*liaX*289*Δ*liaYZ* mutant transformed with pMSP3535 and derivatives containing *liaY* and *liaYZ*. Scale bar (white) at 2 µm

(B) Quantification of septal localization of anionic phospholipid microdomains with NAO staining in *E. faecalis* OG1RFΔ*liaX*289*Δ*liaYZ* mutant transformed with pMSP3535 and derivatives containing *liaY* and *liaYZ*. **p<0.001 via one way ANOVA with multiple comparisons

Whole images were adjusted for “Black Balance” per BZ-X800 Image Analysis Software with individual representative selected.

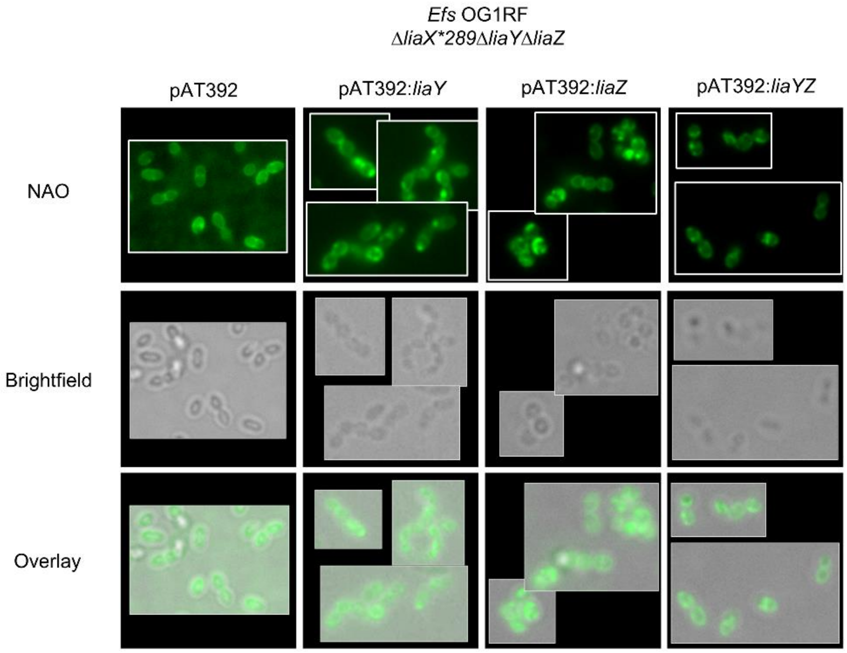

Supplementary Figure 13: Additional Images for liaYZ *trans-complementation*
Evaluation of anionic phospholipid domains using NAO staining (top row) in deletions of *liaYZ* mutants complemented with pAT392::*liaYZ* with brightfield image (middle row) and overlay (bottom row). Whole images were adjusted for “Black Balance” per BZ-X800 Image Analysis Software.

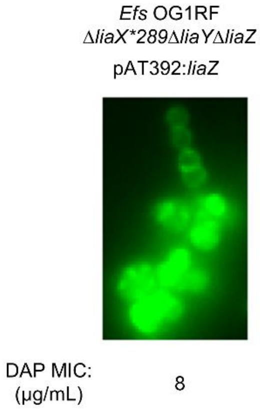

Supplementary Figure 14. trans expression of liaZ *causes clumping phenotype*
Representative image of "clumping" phenotype of cells expressing *liaZ* from pAT392 and stained with NAO. Whole images were adjusted for "Black Balance" per BZ-X800 Image Analysis Software with individual representative selected.

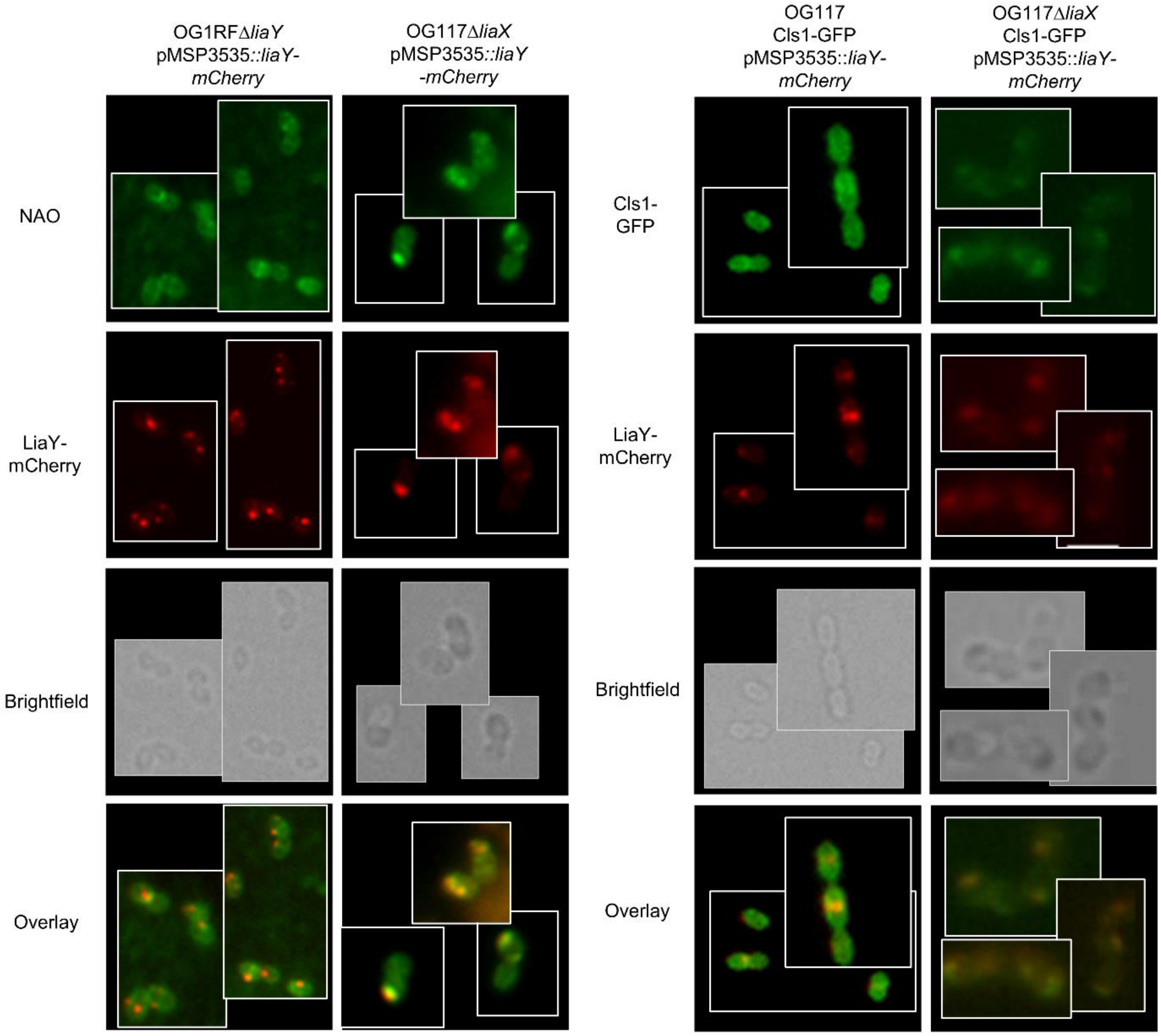

Supplementary Figure 15: Additional Images of LiaY and Cls and NAO

Evaluation of anionic phospholipid domains using NAO staining (top row) in strains listed with mCherry fluorescence (second row), brightfield image (third row) and overlay (bottom row). Whole images were adjusted for “Black Balance” per BZ-X800 Image Analysis Software.

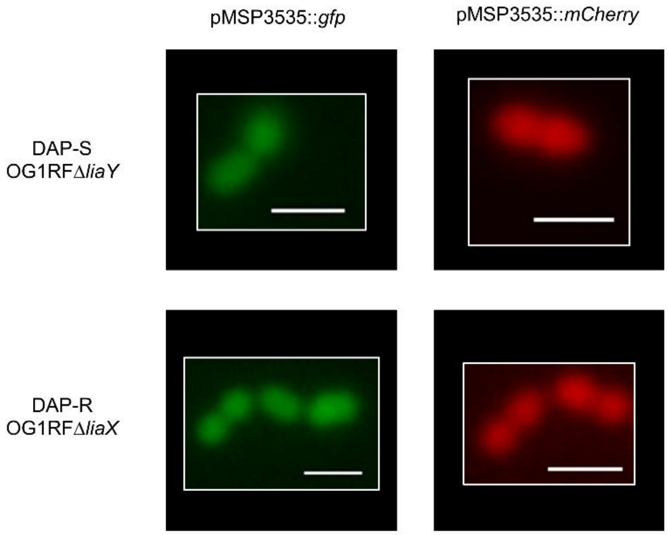

Supplementary Figure 16: Fluorescent tags do not self-aggregate.
Genes encoding GFP or mCherry were expressed on pMSP3535 in representative DAP-S *Efs* OG1RFΔ*liaY* and DAP-R *Efs* OG1RFΔ*liaX,* followed by visualization via fluorescence microscopy showing only a diffuse pattern with no discrete foci. Scale bar (white) at 2µm.
Whole images were adjusted for “Black Balance” per BZ-X800 Image Analysis Software with individual representative selected.

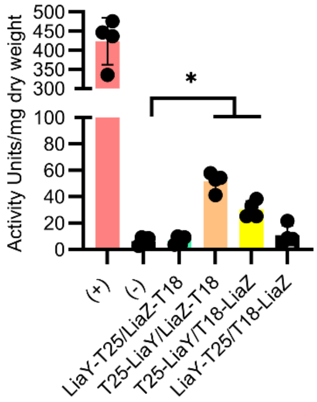

Supplementary Figure 17. LiaY interacts with LiaZ.
LiaY-LiaZ interactions were screened via the bacterial two hybrid system. Proteins were tagged at either the N- or C-terminus, co-transformed into *E. coli* BTH101 and activity recorded via a beta galactosidase assay. A leucine zipper interaction was used as the positive control (T18-zip/T25-zip, green bar) with two non-tagged empty vectors used as negative controls (T18/T25). A positive interaction was identified between LiaY and LiaZ when LiaY was tagged at the N-terminus (T25-LiaY). Results represent the average of 3-5 experiments. *$p<0.05$ via individual t-tests against (-)

Supplementary Table 1. Enterococcus faecalis strains used in this study

Strain	DAP MIC (µg/mL)	Characteristics	Reference(s)
OG1RF	2	Laboratory strain	Lab strain
OG1RFΔ*liaX*	12	OG1RF containing a full deletion of *liaX*	4
OG1RF*liaX**289	12	OG1RF containing a frameshift causing a truncation of predicted LiaX at amino acid position 289	4
OG1RF*liaX**289Δ*liaZ*	8	OG1RF *liaX**289 with a deletion of *liaZ*	This work
OG1RF*liaX**289Δ*liaYZ*	6	OG1RF *liaX**289 with deletions of *liaY* and *liaZ*	This work
OG1RF*liaX**289Δ*liaYZ*(pMSP3535)	8	OG1RF *liaX**289Δ*liaYZ* containing nisin-inducible pMSP3535 vector with no inserts	This work
OG1RF*liaX**289Δ*liaYZ* (pMSP3535::*liaY*)	24	OG1RF*liaX**289Δ*liaYZ* expressing *liaY* on pMSP3535 plasmid under the control of the nisin-inducible *nis* promoter.	This work
OG1RF*liaX**289Δ*liaYZ* pMSP3535::*liaYZ*	24	OG1RF*liaX**289Δ*liaYZ* expressing both *liaY* and *liaZ* on pMSP3535 plasmid under the control of the nisin-inducible *nis* promoter.	This work
OG1RF*liaX**289Δ*liaYZ*(pAT392)	4	OG1RF*liaX**289Δ*liaYZ* containing plasmid pAT392 with no inserts.	This work
OG1RF*liaX**289Δ*liaYZ* pAT392::*liaY*	8	OG1RF*liaX**289Δ*liaYZ* expressing *liaY* on pAT392 plasmid under the control of the P2 promoter.	This work
OG1RF*liaX**289Δ*liaYZ* pAT392::*liaYZ*	8	OG1RF*liaX**289Δ*liaYZ* expressing both *liaY* and *liaZ* on plasmid pAT392 under the control of the P2 promoter.	This work
OG117	1.5 - 2	Derivative of laboratory strain *Efs* OG1RF containing constitutively expressed *cas9* for use with CRISPR-Cas9 mutagenesis in enterococci. Representative DAP-sensitive strain	18
OG117Δ*cls1*	1	OG117 containing a deletion of *cls1*	This work
OG117Δ*cls2*	1	OG117 containing a deletion of *cls2*	This work
OG117Δ*cls1*Δ*cls2*	1.5	OG117 containing deletions of *cls1* and *cls2*	This work

OG117Δ*liaX*	8-12	OG117 containing a full deletion of *liaX*. Representative DAP-R strain with an activated LiaFSR system	This work
OG117Δ*liaX*Δ*cls1*	8	OG117Δ*liaX* containing a deletion of *cls1*	This work
OG117Δ*liaX*Δ*cls2*	12	OG117Δ*liaX* containing a deletion of *cls2*	This work
OG117Δ*liaX*Δ*cls1*Δ*cls2*	4	OG117Δ*liaX* containing deletions of *cls1* and *cls2*	This work
OG117Δ*liaX*Δ*cls1*Δ*cls2*(pAT392)	4	OG117Δ*liaX*Δ*cls1*Δ*cls2* containing plasmid pAT392 vector with no inserts.	This work
OG117Δ*liaX*Δ*cls1*Δ*cls2*(pAT392::*cls1*)	24	OG117Δ*liaX*Δ*cls1*Δ*cls2* containing *cls1* expressed from pAT392 vector under the control of the P2 promoter.	This work
OG117Δ*liaX*Δ*cls1*Δ*cls2*(pAT392::*cls2*)	16	OG117Δ*liaX*Δ*cls1*Δ*cls2* containing *cls2* expressed from pAT392 vector under the control of the P2 promoter.	This work
OG117Δ*liaX*Δ*cls1*Δ*cls2*(pMSP3535)	4	OG117Δ*liaX*Δ*cls1*Δ*cls2* containing nisin-inducible pMSP3535 plasmid with no inserts.	This work
OG117Δ*liaX*Δ*cls1*Δ*cls2*(pMSP3535::*cls1*)	16	OG117Δ*liaX*Δ*cls1*Δ*cls2* containing *cls1* expressed on pMSP3535 plasmid under the control of the nisin-inducible *nis* promoter.	This work
OG117Δ*liaX*Δ*cls1*Δ*cls2*(pMSP3535::*cls2*)	8	OG117Δ*liaX*Δ*cls1*Δ*cls2* containing *cls2* expressed on pMSP3535 plasmid under the control of the nisin-inducible *nis* promoter	This work

DAP, daptomycin; MIC, minimal inhibitory concentrations.

Supplementary Table 2. Primers used in this study

Primer	Sequence 5' → 3'	Description (F, forward. R, reverse)
**Deletion of *cls1***		
FrA_*cls1*_F	atattacagctccagatccatatccttcttCAAAATTGGATCAAAAAGAA	F, lowercase: plasmid, uppercase: fragment upstream of *cls1*
FrA_*cls1*_R	TCATTTTGTGAAAAACAGTTTTGTCTTCCTCCTTTATTTGTTA	R, upstream of *cls1*
FrB_*cls1*_F	CAAATAAAGGAGGAAGACAAAACTGTTTTTCACAAAATGACG	F, downstream of *cls1*
FrB_*cls1*_R	gaagcgaaaaaggagaagtcggttcagaaaAAGACTGTCCACATTATGTTGC	R, lowercase: plasmid, uppercase: fragment downstream of *cls1*
Spacer_*cls1*_F	GTTTTGAACTTTATAGTCAAAAGATGCCGAgttttagagtcatgttgtttagaatgg	F, lowercase: plasmid, uppercase: spacer sequence within *cls1*
Spacer_*cls1*_R	TCGGCATCTTTTGACTATAAAGTTCAAAACtttcattgctattatacccatgtag	F, lowercase: plasmid, uppercase: spacer sequence within *cls1*
**Deletion of *cls2***		
FrA_*cls2*_F	agctccagatccatatccttcttACCCAAGTGATTACGATTGAC	F, lowercase: plasmid, uppercase: fragment upstream of *cls2*
FrA_*cls2*_R	CTGATTTATTGACGCTTACCTCCTTCTTACTTC	R, upstream of *cls2*
FrB_*cls2*_F	AGGAGGTAAGCGTCAATAAATCAGCAGTGAATG	F, downstream of *cls2*
FrB_*cls2*_R	cgaaaaaggagaagtcggttcagaaaTCTGGAATGACTTTTTCCAA	R, lowercase: plasmid, uppercase: fragment downstream of *cls2*
Spacer_*cls2*_F	TTACTGGCGAGATACCCATATTCGTTTGGTgttttagagtcatgttgtttagaatgg	F, lowercase: plasmid, uppercase: spacer sequence within *cls2*
Spacer_*cls2*_R	ACCAAACGAATATGGGTATCTCGCCAGTAAtttcattgctattatacccatgtag	F, lowercase: plasmid, uppercase: spacer sequence within *cls2*
**Other *cls1* constructs**		
BamHI_*cls1*_pAT_F	CGGGATCCTAACAAATAAAGGAGGAAGACAATTG	F, for cloning *cls1* into pAT392, containing BamHI site
XbaI_*cls1*_pAT_R	CGTCTAGATTAAAGAATTGGTGAAAATAATCGTG	R, for cloning *cls1* into pAT392, containing XbaI site
*cls1-gfp*_FrA_F	CTCCAGATCCATATCCTTCTTGGATCATATCGGTACCAAAA	F, for construction of chromosomal insertion of GFP tag to Cls1 via CRISPR.
*cls1-gfp*_FrA_R	AATTTGTAACTTCGTATATCTTGATTG	R, for construction of chromosomal insertion of GFP tag to Cls1 via CRISPR
*cls1-gfp*_FrA2_F	AATCAAGATATACGAAGTTACAAATTAAAC	F, for construction of chromosomal insertion of GFP tag to Cls1 via CRISPR

*cls1-gfp*_FrA2_R	ACTACTGCCACCAAGAATTGG TGAAAATAATCGTG	R, for construction of chromosomal insertion of GFP tag to Cls1 via CRISPR
*cls1-gfp*_GFP_F	CAATTCTTGGTGGCAGTAGTA AAGGAGAAGAACTTTTC	F, for construction of chromosomal insertion of GFP tag to Cls1 via CRISPR
*cls1-gfp*_GFP_R	GTGAAAAACAGTTTTATTTGTA TAGTTCATCCATG	R, for construction of chromosomal insertion of GFP tag to Cls1 via CRISPR
*cls1-gfp*_FrB_F	GAACTATACAAATAAAACTGTT TTTCACAAAATGA	F, for construction of chromosomal insertion of GFP tag to Cls1 via CRISPR
*cls1-gfp*_FrB_R	AAGGAGAAGTCGGTTCAGAAA AAGACTGTCCACATTATGTT	R, for construction of chromosomal insertion of GFP tag to Cls1 via CRISPR
*cls1-gfp*_spacer_F	GCATTTGCTTCAAAGTTTAATT TGTAACTTGTTTTAGAGTCATG TTGTTTAG	F, for construction of chromosomal insertion of GFP tag to Cls1 via CRISPR
*cls1-gfp*_spacer_R	AAGTTACAAATTAAACTTTGAA GCAAATGCTTTCATTGCTATTA TACCCATGTAG	R, for construction of chromosomal insertion of GFP tag to Cls1 via CRISPR
**Other *cls2* constructs**		
BamHI_*cls2*_pmsp_F	CGGGATCCTAATCTTAGTATG AAGTAAGAAGGAGGTAAGCG	F, for cloning *cls2* into pMSP3535 containing BamHI site
XbaI_*cls2*_pmsp_R	GCTCTAGATTACAAGACTGGT GACAACAAGCG	R, for cloning *cls2* into pMSP3535 containing XbaI site
BamHI_*cls2*_pat_F	TCGGTACCCGGGGATCCTCTT AGTATGAAGTAAGAAGGAGGT AAGCG	F, for cloning *cls2* into pAT392 containing BamHI site
XbaI_*cls2*_pat_R	GCAAGGGGAATTGACTCTAGA TTACAAGACTGGTGACAACAA GCG	R, for cloning *cls2* into pAT392 containing BamHI site
*gfp-cls2_cls2*_F	ACAAAGGTGGCAGTAAAATTT TTGTTTGGATTTTG	F, for cloning gfp-cls2 into pMSP3535
*gfp-cls2_cls2*_R	AGACCGGCCTCGAGTCTAGAT TACAAGACTGGTGACAAC	R, for cloning gfp-cls2 into pMSP3535
*gfp-cls2_GFP*_F	TCTTGGGTGGCAGTAGTAAAG GAGAAGAACTTTTC	F, for cloning gfp-cls2 into pMSP3535
*gfp-cls2_GFP*_R	AATTTTACTGCCACCTTTGTAT AGTTCATCCATGC	R, for cloning gfp-cls2 into pMSP3535
**Deletion of *liaY***		
FrA_*liaY*_F	CGGGATCCGACATTGATGTGG ATGATGAAA	F, fragment upstream of *liaY.* BamHI site
FrA_*liaY*_R	CAATCGCTGAAAATATGTCATA TGTTTTCACCTCGTCTAC	R, fragment upstream of *liaY.*
FrB_*liaY*_F	ATGACATATTTTCAGCGATTG GTTGTTAATACACTGACAT	F, fragment downstream of *liaY.*
FrB_*liaY*_R	CGGAATTCAATCAAGGACGAG AACCAATC	R, fragment downstream of *liaY.* EcoRI site
**Deletion of *liaYZ***		

FrA_*liaYZ*_F	GCTCTAGAGACATTGATGTGG ATGATGAAA	F, fragment upstream of *liaYZ.* XbaI site
FrA_*liaYZ*_R	ATGTTTTCACCTCATCTACTAA ATTTTACACTAGAAAGGC	R, fragment upstream of *liaYZ.*
FrB_*liaYZ*_F	AGTAGATGAGGTGAAAACATA AAAAGCCTGAAGAACGTAGCA	F, fragment downstream of *liaYZ.*
FrB_*liaYZ*_R	CGGGATCCATCGGCCGCGCT ACAATCGC	R, fragment downstream of *liaYZ.* BamHI site
**Other *liaY* constructs**		
BamHI_*liaY*_F	CGGGATCCACGAGGTGAAAA CATATGAAAAG	F, upstream of *liaY* start codon BamHI site
XbaI_*liaY*_R	GCTCTAGATTAAAAGTCACTC CATTCGTCATC	R, 3' end of *liaY* including stop codon. XbaI site
*liaY-mCherry_liaY*_F	CAGGAGACTCTGCATGGATCC TAGTAGACGAGGTGAAAACAT ATG	F, for cloning of liaY-mCherry into pMSP3535, contains BamHI site
*liaY-mCherry_liaY*_R	CTTGGAAACGCTTCCGCCACA ACATCCTGGACAACAGC	R, for cloning of liaY-mCherry into pMSP3535
*liaY-mCherry_mCherry*_F	GTGGCGGAAGCGTTTCCAAG GGCGAGGAGG	F, for cloning of liaY-mCherry into pMSP3535
*liaY-mCherry_mCherry*_R	AGACCGGCCTCGAGTCTAGAT TATTTGTACAGCTCATCCATGC C	R, for cloning of liaY-mCherry into pMSP3535, contains XbaI site
*liaY-mCherry*_Q5_F	GTTTCCAAGGGCGAGGAG	F, generation of pMSP3535 mCherry control through Q5 mutagenesis
*liaY-mCherry*_Q5_R	CATATGTTTTCACCTCGTCTAC	R, generation of pMSP3535 mCherry control through Q5 mutagenesis
**Complementation of *liaYZ***		
BamHI_*liaYZ*_F	CGGGATCCACGAGGTGAAAA CATATGAAAAG	F, upstream of *liaY* start codon BamHI site
XbaI_*liaYZ*_R	GCTCTAGATTATTCATATTTCG ATAAATTGTG	R, 3' end of *liaZ* including stop codon. XbaI site
**Bacterial Two Hybrid Studies**		
*cls1*_F	CGCGGATCCGATTCTTAGCGT CTTAACAGT	F, for cloning *cls1* into pKT25/pKNT25 or pUT18/pUT18C
*cls1*_R	CCGGAATTCAGAATTGGTGAA AATAATCGT	R, for cloning *cls1* into pKNT25/pUT18
*cls1*_R2	CCGGAATTCTTTAAAGAATTG GTGAAAAT	R, for cloning *cls1* into pKT25/pUT18C, contains stop codon
*cls2*_F	CGCGGATCCGAAAATTTTTGT TTGGATTTT	F, for cloning *cls2* into pKT25/pKNT25 or pUT18/pUT18C
*cls2*_R	CCGGAATTCAAGACTGGTGAC AACAAGC	R, for cloning *cls2* into pKNT25/pUT18
*cls2*_R2	CCGGAATTCATTACAAGACTG GTGACAACA	R, for cloning *cls2* into pKT25/pUT18C, contains stop codon
*liaY*_F	CGCGGATCCGAAAAGAAAACT AACAAAATCTCCG	F, for cloning *liaY* into pKT25/pKNT25 or pUT18/pUT18C

*liaY*_R	CCGGAATTCAAGTCACTCCAT TCGTCATC	R, for cloning *liaY* into pKNT25/pUT18
*liaY*_R2	CCGGAATTCATTAAAAGTCAC TCCATTCGTCAT	R, for cloning *liaY* into pKT25/pUT18C, contains stop codon
*liaZ*_F	CGCGGATCCGACATATTTTCA GCGATTGGT	F, for cloning *liaZ* into pKT25/pKNT25 or pUT18/pUT18C
*liaZ*_R	CCGGAATTCTCATATTTCGATA AATTGTGAT	R, for cloning *liaZ* into pKNT25/pUT18
*liaZ*_R2	CCGGAATTCTTTTATTCATATT TCGATAAATTGTG	R, for cloning *liaZ* into pKT25/pUT18C, contains stop codon
**qRT-PCR**		
*16S*_F	GGAGACTTGAGTGCAGAAGA	F, Housekeeping gene, 16S rRNA of OG1RF
*16S*_R	CGTCAGTTACAGACCAGAGAG	R, Housekeeping gene, 16S rRNA of OG1RF
*gyrB*_F	AAAAGGCATGTTGGCTTCAAA	F, Housekeeping gene, *gyrB* of OG1RF
*gyrB*_R	GCTTCCCTGGCAAGTTGCTA	R, Housekeeping gene, *gyrB* of OG1RF
*cls1*_F	GGTGCCAGAGGCAAATACAT	F, to amplify *cls1* for qRTPCR
*cls1*_R	AATACGGCGTTTGAATCCAG	R, to amplify *cls1* for qRTPCR
*cls2*_F	GTTGTTAGTGGCGGTTCGTT	F, to amplify *cls2* for qRTPCR
*cls2*_R	TTAATGCCGCGTTTGTGTAA	R, to amplify *cls2* for qRTPCR

***Supplementary Table 3:* liaS *mutations in* Efs *OG1RF*ΔliaX*289ΔliaY**

Gene	Predicted Change
liaS	Ala316→Thr

Supplementary Table 4: Reagents

Reagent	Source	Catalog Number
Luria Bertani Broth/Agar	BD	244620/244520
Brain Heart Infusion Broth/Agar	BD	237500/241830
Tryptic Soy Broth	BD	B257543
Erythromycin	Sigma-Aldrich	E5389
Gentamicin	Sigma-Aldrich	G3632
Daptomycin	Morris& Dickson	650903
Chloramphenicol	Sigma-Aldrich	C1919
Nisin	Sigma-Aldrich	N5764
Spectinomycin	Sigma-Aldrich	S4014
Kanamycin	Sigma-Aldrich	K1377
Ampicillin	Sigma-Aldrich	A9393
IPTG	GoldBio	I2481C
o-nitrophenol-β-galactoside	Sigma-Aldrich	N1127
Sodium carbonate	Fisher	S263-500
Toluene	FIsher	T324-500
10% SDS solution	Fisher	15553027
p-chloro-phenylalanine	Sigma-Aldrich	C6506
Phusion	New England BioLabs	M0530

PrimeStar Max	Takara Bio	R045B
Gibson Assembly Cloning Kit	New England BioLabs	E5510S
GoTaq DNA Polymerase	Promega	M3001
E-test (Daptomycin)	Biomerieux	412323
10-N-nonyl acridine orange	Invitrogen	A1372
TC-ReAsH™ II In-Cell Tetracysteine Tag Detection Kit	Invitrogen	T34562
Bacterial Two Hybrid Kit	Euromedex	EUK001
Chloroform	FisherChemical, Optima	C2974
Methanol	FisherChemical, Optima	A456-4
Acetonitrile	FisherChemical, Optima	A996SK-4
PE 15:0/15:0	Avanti Polar Lipids	850704
